# Supplementary material for: Integrated Analysis of Methylomic and Transcriptomic Data to Identify Potential Diagnostic Biomarkers for Major Depressive Disorder
Source: Genes (Basel). 2021 Jan 27;12(2):178. doi: 10.3390/genes12020178 (PMC7912210; doi:10.3390/genes12020178)
Supplement: Supplementary file 1 [file genes-12-00178-s001.zip › Table S1-S10/Table S1-S6.docx]

**Table S1**: Demographic and clinical features for GSE113725

**Table S2**: Demographic and clinical features for GSK-HiTDiP and Janssen-BRC (GSE98793) case-control studies

**Table S3**: Gene symbol of 1056 DEGs

**Table S4**: Gene symbol of 8313 DMGs

**Table S5**: Pathways the 46 hypo-up genes are involved in

**Table S6**: Pathways the 71 hyper-down genes are involved in

**Table S1** Demographic and clinical features for GSE113725

| Group | depression N=100 | healthy control N=50 |
| --- | --- | --- |
| Female | 74 | 37 |
| Male | 26 | 13 |
| Mean Age | 45.5 | 45.4 |
| Smoker | 6 | 2 |
| Mean BMI | 25.9 | 25.8 |
| Anti-depressant | 57 | no |
| Ethnicity | white ethnicity | |

**Inclusion Criteria:** Has been diagnosed with depression and required regular medical treatment.

**Exclusion Criteria:** not mention.

**Table S2** Demographic and clinical features for GSK-HiTDiP and Janssen-BRC case-control studies (GSE98793)

| Source | GSK-HiTDIP | | Janssen-BRC | |
| --- | --- | --- | --- | --- |
| Group | Control (N=57) | Depressed (N=113) | Control(N=100) | Depressed(N=94) |
| Female | 16 | 25 | 61 | 58 |
| Male | 41 | 88 | 39 | 36 |
| Age Mean (SD) | 52 (11) | 52 (12) | 38 (15) | 39 (14) |
| BMI Mean | not mention | | 25.5 | 25.6 |
| Ethnicity | Caucasian | | Sydneysiders | |

**Inclusion Criteria** for **GSK-HiTDIP**: at least two episodes of depression satisfying DSM-IV or ICD10 criteria.

**Exclusion Criteria** for **GSK-HiTDIP**: (1) experience of mood-incongruent psychotic symptoms or lifetime history of schizophrenia, schizoaffective disorder or other axis, disorders; (2) lifetime history of intravenous drug use or diagnosis of drug dependency; (3) lifetime history of depression secondary to alcohol, substance abuse, medical disorders or use of prescribed medication; (4) lifetime history of obsessive compulsive disorder (OCD) or post-traumatic stress disorder (PTSD).

**Inclusion Criteria** for **Janssen-BRC**: HAM-D17 total score >= 18.

**Exclusion Criteria** for **Janssen-BRC**: Patients with a history of substance abuse in the last year, or with a history of a mental disorder not related to depression were excluded.

**Note**: The 128MDD and 64 healthy controls in GSE98793 are on a subset of GSK-HiTDIP and Janssen-BRC. The Platform is GPL570 [HG-U133_Plus_2] Affymetrix Human Genome U133 Plus 2.0 Array.

**Table S3.** Gene symbol of 1056 DEGs

| **Direction** | **Gene symbol** |
| --- | --- |
| up-regulated (713) | SEPT4 SEPT10 ABCD1 ABCG2 AC005498.3 AC005838.2 AC010145.3 AC018462.2 AC024560.2 AC068831.3 AC079305.10 ACER1 ACOT1 ACOT2 ACP2 ADORA3 ADRA2A AF067845.1 AFAP1 AHRR AIRE AJAP1 AK023627 AK056098 AK056982 AKAP4 AKR1D1 AL022344.5 AL109706 ALAS2 ALOX12 ALPL ALPP ALPPL2 AMELY ANKRD22 ANO5 AOC1 AP000525.9 AP1M2 APCDD1 ARG1 ARHGAP11B ASB17 ASIC4 ASPH ATP6V0C ATP8B4 AX746670 AX746699 AZU1 B3GNT8 BAI2 BC031952 BC034423 BC034444 BC042091 BC042855 BC063675 BC071802 BC089413 BDKRB1 BEAN1 BMS1P17 BMS1P18 BMX BPIFA3 BPY2 C11orf45 C14orf183 C15orf26 C16orf59 C17orf100 C17orf97 C1orf234 C1RL C20orf85 C3orf43 C5orf15 C5orf30 C7orf63 C9 C9orf172 C9orf66 CABP2 CAMP CAPN8 CAPNS2 CASP12 CATIP-AS1 CBLN1 CCDC108 CCDC151 CCDC170 CCDC64B CCL20 CCL7 CCPG1 CCSER1 CCZ1 CD163 CD177 CDH5 CDK5R1 CDRT15L2 CEACAM4 CEACAM6 CEACAM8 CECR5-AS1 CECR6 CES1 CHIT1 CHRM2 CHRM4 CIDEA CLDN8 CLEC12B CLEC1A CLEC3B CLEC4E CLEC5A CLU CNTLN CNTN6 CNTNAP3 CNTNAP3B CNTNAP3P2 COL28A1 COPA COX7A1 CPA1 CPD CRISP3 CRNN CSRP3 CST1 CT55 CTA-280A3__B.2 CTA-292E10.6 CTAGE11P CTC-203F4.2 CTC-428G20.3 CTD-2313J17.5 CTD-3138B18.5 CTD-3203P2.1 CTF1 CTNNAL1 CTRB2 CTSE CYBA CYBRD1 CYP1B1 DEFA1 DEFA1B DEFA3 DEFA4 DEFB108B DGCR9 DGKI DHDH DHH DHRS7C DIAPH2-AS1 DIRC3 DISC1 DKFZP434F142 DMRTC2 DNAJC6 DOCK4 DPY19L1P1 DSC2 DSCAML1 DUX2 ECHDC3 ECRP EFNA3 EGF EPDR1 EPHX1 ERV3-2 ESAM ESRG EVPLL EXOC6 EXOSC7 F2 F5 FAM107A FAM109B FAM131B FAM181A FAM205A FAM205B FAM209B FAM225A FAM225B FAM71F2 FAM86B3P FAM90A1 FAM90A25P FAM90A2P FBXO2 FBXO6 FECH FERMT3 FGD4 FGF13 FKBP5 FKBP9 FLJ13224 FLJ32154 FLJ36840 FLJ37786 FLJ39080 FNDC1 FNDC7 FOLH1B FOLR3 FOXD2 FPGT FSHB FSTL1 GABRA4 GADD45A GADD45G GAL3ST3 GBAP1 GCG GJB6 GLRX5 GLT1D1 GNA14-AS1 GOT2P1 GP1BA GPR45 GRB10 GRIA1 GRID2 GRINA GTPBP10 GUCY2D GXYLT2 H2AFY H2BFM HAGLROS HBD HCAR1 HECW2 HERC2 HERC2P10 HESX1 HIAT1 HIP1 HK2 HLA-DPA2 HMP19 HOXA1 HP HPR HPSE HRASLS HSD3B7 HSF4 HTR7 HTR7P1 HTRA1 HTRA4 HUS1B HYAL3 HYPM ICAM5 IFI44 IFT81 IGHV3-73 IGLVI-70 IL13RA1 IL1R1 INHBA INPP5J INSC INTU IRAK3 IRGC IRS4 IRX1 ITGA2B ITGA3 ITGB5 JARID2-AS1 KATNAL2 KCNE1 KCNK15 KDM8 KEL KIAA0319L KIAA1598 KIAA1875 KIR2DS3 KIR2DS4 KL KREMEN1 KRT37 KRTAP8-1 LAD1 LCN2 LECT2 LGSN LHFPL2 LILRA6 LINC00173 LINC00221 LINC00266-1 LINC00284 LINC00305 LINC00459 LINC00487 LINC00566 LINC00616 LINC00639 LINC00654 LINC00673 LINC00880 LINC00892 LINC00909 LINC00933 LINC00968 LINC01082 LINC01093 LINC01102 LINC01226 LINC01271 LINC01279 LINC01281 LINC01289 LINC01350 LINC01352 LINC01362 LINC01410 LINC01449 LMO7DN LOC100128185 LOC100287808 LOC100288123 LOC100288637 LOC100505774 LOC100506119 LOC100506289 LOC100506470 LOC100506675 LOC100506791 LOC100507670 LOC100510710 LOC100653057 LOC100653086 LOC101060004 LOC101926916 LOC101927085 LOC101927157 LOC101927248 LOC101927278 LOC101927830 LOC101928222 LOC101928290 LOC101928317 LOC101928457 LOC101928521 LOC101928631 LOC101928669 LOC101928707 LOC101928708 LOC101928858 LOC101929004 LOC101929007 LOC101929047 LOC101929269 LOC101929312 LOC101929657 LOC101929687 LOC101929832 LOC101930100 LOC101930149 LOC101930286 LOC102723742 LOC102723918 LOC102723990 LOC102725382 LOC221814 LOC283588 LOC284240 LOC284933 LOC286052 LOC339666 LOC340178 LOC401098 LOC439994 LOC440864 LOC440934 LOC497256 LOC642361 LOC643355 LOC644450 LOC646778 LOC653486 LOC653581 LOC654780 LPP-AS2 LRFN1 LRG1 LRRC37A5P LRRC4C LTBP1 LTF LURAP1L MAFB MAGEA4 MAGEA6 MAGEC1 MALL MAOA MBOAT2 MCEMP1 MED14OS MED15P9 MEF2C-AS1 MEFV MEG8 MGST1 MIR31HG MIR424 MIR4435-1HG MIR4454 MIR503 MIR503HG MIR6732 MIRLET7BHG MMP10 MMP3 MMP7 MMP8 MMP9 MMRN1 MORN2 MPO MSH6 MTNR1B MYBPC2 MYCNUN MYEOV MYF6 MYH4 NAIP NAV2-IT1 NCEH1 NCOA6 NEU4 NINL NIPAL1 NKX2-1 NLRP6 NLRP9 NOV NRG1 NRGN NRSN1 ODAM ODF3L2 OIT3 OLAH OLFM4 OLFML2A OLFML2B OLIG1 OLR1 OR1D5 OR1G1 OR51J1 ORM1 ORM2 OSCAR OVOL3 P2RX5 P2RX5-TAX1BP3 PADI4 PAGE1 PANX3 PATE1 PCDHB12 PCDHB5 PCDHGB8P PDE6H PEAR1 PERM1 PF4V1 PGLYRP1 PGM5 PHACTR3 PHOSPHO1 PIWIL4 PLA2G7 PLAC8L1 PLBD1 PLEKHM2 PLOD1 PLTP PLXNA2 PNMT POM121 PP12613 PP13 PRKAR2B PRO2012 PROS1 PRSS53 PRTN3 PTGES3L PVRL2 R3HDML RAB42 RAI2 RBSG3 RETN RETNLB RGAG1 RHCE RHD RHOBTB1 RN7SKP150 RNASE1 RNASE3 RNASE4 RNF212B RORC RP11-118G23.1 RP11-13K12.5 RP11-164P12.4 RP11-171I2.2 RP11-173M1.8 RP11-181E10.3 RP11-199F11.2 RP11-231E19.1 RP11-24P14.1 RP11-266L9.1 RP11-278J20.2 RP11-305O6.3 RP11-332H18.4 RP11-343H5.6 RP11-357G3.1 RP11-359E8.5 RP11-389C8.2 RP11-394I13.2 RP11-443B7.1 RP11-457I16.2 RP11-457K10.1 RP11-474D1.2 RP11-476D10.1 RP11-524D16__A.3 RP11-540O11.1 RP11-573N10.1 RP11-5N11.2 RP11-6I2.3 RP11-752D24.2 RP1-179N16.6 RP1-192P9.1 RP11-966I7.2 RP11-981G7.6 RP3-525N10.2 RP4-730D4.1 RP4-758J24.5 RP5-1103G7.10 RP5-968J1.1 RPS11P6 RRAGD RSPH3 RSPH9 S100A12 S100A3 S100A7 S100P SATB2-AS1 SCART1 SCGB1C1 SCN1B SCN5A SDHD SDR9C7 SEC14L4 SELP SEPSECS-AS1 SERPINB2 SERPING1 SERPINI2 SH3TC2 SHBG SIAH2 SIPA1L2 SIRPAP1 SLC1A3 SLC22A1 SLC22A14 SLC22A15 SLC22A16 SLC24A3 SLC26A4 SLC26A8 SLC26A9 SLC2A1 SLC30A3 SLC35D3 SLC4A2 SLC5A5 SLC8A3 SLPI SMIM1 SMIM2 SMIM5 SNAI1 SNORA5B SNORD112 SORT1 SOX14 SPANXA1 SPARC SPATA42 SPATC1L SPHK1 SPRR2C ST6GALNAC3 STAC STEAP1 STK24-AS1 SUCNR1 SULT1B1 SYT6 TAS1R2 TAS2R13 TAS2R16 TAX1BP3 TBCC TBL1Y TBRG4 TCEAL4 TCFL5 TCN1 TCN2 TCTE3 TDRD9 TEC TENC1 TENM2 TFF3 THAP8 THBS2 THRB TLR5 TLR8-AS1 TMC1 TMEM45A TMEM45B TMEM52B TMEM53 TMEM72-AS1 TMEM86B TMLHE-AS1 TMX4 TNFAIP6 TNFSF13 TNNI1 TNNT1 TPST1 TREML1 TRIM71 TSHZ3 TSKS TSNAX-DISC1 TSPO2 TSPY1 TSPY10 TSPY3 TSPY4 TSPY8 TTC26 TTC32 TTC36 TTC7B TTLL13 TTN TTTY11 TUBB2B TUBB8P2 TULP2 UBBP1 UBBP2 UBBP4 UBE2S UBTD1 UMOD VEGFC VEPH1 VNN1 VSTM1 VTCN1 WASF1 WNT8B XAGE3 ZC3H12A ZIC2 ZNF117 ZNF423 ZNF513 ZNF607 ZNF705G ZNF77 ZNF788 |
| down-regulated  (793) | SEPT1 A2M-AS1 A2MP1 ABCD2 abParts AC005224.2 AC007680.2 AC009237.8 AC083843.1 AC090627.1 AC139100.2 ADAMTS6 ADRA1B AF086126 AF086294 AF131215.3 AF131215.4 AF131215.8 AF198444 AGAP4 AIF1L AJ420595 AJ606316 AK055910 AK055967 AK093356 AK096592 AK123826 AK291611 AKAP3 AKR1C3 AL133493.2 ALDH1L1-AS2 ALKBH3-AS1 ANGPTL7 ANKRD20A1 ANKRD20A12P ANKRD20A2 ANKRD20A3 ANKRD20A4 ANKRD20A5P ANKRD20A8P ANKRD26 ANKRD34A ANKRD35 ANKRD44-IT1 APBA1 AQP11 ARIH2OS ATF7IP2 ATP1A3 AV2S1A1 AV8S2 BC031864 BC039319 BC040734 BC041025 BC043223 BC045560 BLK BMS1P6 BOLA3 BPIFA1 BPIFB9P BRDT BTBD3 BTN3A2 BX538226 C10orf95 C11orf63 C11orf74 C12orf29 C14orf142 C14orf28 C15orf54 C16orf52 C16orf78 C17orf47 C1orf112 C1orf204 C20orf57 C4A C4B C4B_2 C6orf195 C6orf201 C7orf33 C8orf15 C8orf59 CALHM1 CALY CARF CATSPERB CBX3P2 CCDC33 CCDC7 CCDC71 CCR5 CCT2 CD1C CD3G CD40LG CD8A CDC37L1 CDCA8 CDH12 CENPK CFLAR-AS1 CHAC2 CHFR CHRM3-AS2 CKAP2 CKB CLECL1 CLUHP3 CLYBL-AS2 CMC1 CNR2 CNTD1 COA4 COMMD8 COPG2IT1 CPA4 CRYGS CSNK2A1 CST8 CST9L CTC-523E23.1 CTD-2002J20.1 CTD-2540F13.2 CTD-2553C6.1 CTD-2561B21.11 CTD-2619J13.13 CTRB1 CTRB3 CXCL13 CYP17A1 CYP26A1 CYP2C19 CYP4F3 DBIL5P DCAF16 DDIT4L DDX11 DDX12P DFNA5 DFNB59 DKFZP434L187 DLG3-AS1 DNAAF2 DNASE1L3 DPPA5 DUSP15 DUSP5 DUX1 DUX3 DUX5 EBF1 EGR3 EIF2A EIF2B3 ELFN1 ENPP3 ENTPD3-AS1 EOMES ERAP2 ERICH3-AS1 EXTL2 FA2H FAM118A FAM175A FAM187B FAM226A FAM226B FAM229B FAR1P1 FAR2P2 FAR2P3 FCER1A FGF9 FGG FKTN FLVCR1-AS1 FMO4 FRMD6-AS1 FRMPD3 FUT8 GALNT12 GAS5-AS1 GCK GCNT4 GEMIN6 GIMAP7 GKN2 GLDC GNMT GOPC GPM6A GPR171 GPR174 GPR18 GPR52 GRHL2 GRID1-AS1 GRPR GSTA4 GUF1 GZMA GZMK H19 HACE1 HAPLN4 HAUS3 HAX1 HCG4 HEATR5B HIST1H4A HLA-DPB1 HLA-DQA1 HLA-DQA2 HLA-DQB1 HLTF HOOK1 HOXC10 HOXD11 HPD hsa-let-7a-3 hsa-let-7b hsa-mir-4763 HSD17B7P2 HSPA2 HSPB2 IBA57-AS1 ICOS ID2 ID2B IFNA21 IFT140 IFT172 IFT80 IGH IGHA1 IGHA2 IGHD IGHG1 IGHG3 IGHG4 IGHM IGHV3-23 IGHV3-54 IGHV4-31 IGHV5-78 IGK IGKC IGKV4-1 IGLC1 IGLJ3 IGLV2-5 IGLV3-19 IKZF3 IL23A IL3RA IL7 IL7R IL9 ILF3-AS1 INHBE INSL6 INTS7 IPW IRAK1BP1 IRGM ISM1-AS1 JAKMIP2-AS1 KANK3 KCNA3 KCNA7 KIAA0125 KIF2B KIRREL3 KLF2 KLHDC4 KLHDC9 KLRB1 KLRC1 KLRC2 KLRC4 KLRC4-KLRK1 KLRG1 KLRK1 KRBOX4 KRMP1 KRT19P2 KRTAP4-1 LAX1 LDLRAD4-AS1 LEPR LHFPL1 LHFPL3-AS2 LIG1 LINC00086 LINC00087 LINC00112 LINC00167 LINC00280 LINC00421 LINC00494 LINC00540 LINC00561 LINC00630 LINC00691 LINC00865 LINC00965 LINC00996 LINC01015 LINC01018 LINC01149 LINC01153 LINC01197 LINC01260 LINC01293 LINC01310 LINC01354 LINC01355 LINC01405 LIPT1 LOC100127974 LOC100128644 LOC100128843 LOC100128993 LOC100129112 LOC100129917 LOC100129935 LOC100130428 LOC100133131 LOC100134445 LOC100287221 LOC100289495 LOC100293211 LOC100505622 LOC100505710 LOC100506235 LOC100506325 LOC100506639 LOC100507330 LOC100507516 LOC100509457 LOC100996654 LOC100996747 LOC101927081 LOC101927137 LOC101927159 LOC101927164 LOC101927348 LOC101927417 LOC101927620 LOC101927641 LOC101927841 LOC101927901 LOC101927972 LOC101928102 LOC101928162 LOC101928221 LOC101928231 LOC101928303 LOC101928403 LOC101928420 LOC101928464 LOC101928496 LOC101928560 LOC101928625 LOC101928703 LOC101928784 LOC101928877 LOC101929002 LOC101929050 LOC101929272 LOC101929325 LOC101929529 LOC101929549 LOC101929631 LOC101929709 LOC101929855 LOC101929876 LOC101929988 LOC101930404 LOC101930405 LOC101930415 LOC102723678 LOC102723692 LOC102723709 LOC102723932 LOC102724156 LOC102724487 LOC102724532 LOC102724718 LOC102724809 LOC102724851 LOC102725383 LOC146795 LOC153684 LOC200830 LOC219690 LOC283038 LOC283922 LOC284513 LOC284926 LOC284930 LOC285095 LOC285300 LOC285628 LOC285902 LOC286190 LOC338620 LOC339988 LOC389831 LOC389834 LOC400965 LOC401068 LOC401913 LOC440173 LOC441052 LOC441454 LOC554206 LOC642846 LOC644135 LOC645188 LOC729164 LOC730202 LOC730961 LOC731157 LOC732360 LPA LRRC3B LRRC66 LRRTM1 LTV1 LUZP2 LYZL6 MAFIP MAP1LC3C MAP3K14-AS1 MAP3K15 MARS2 MCM10 MCM2 MED21 METTL21EP MGC15705 MGC40069 MGC45800 MIR146A MIR155 MIR155HG MIR205 MIR492 MIR675 MMP20 MOCS3 MPRIP-AS1 MRGPRX4 MRPL13 MRPL45P2 MRPS28 MS4A1 MS4A7 MTHFD1L MYOM1 NAG18 NANOS1 NCR3LG1 NELL2 NFE2L2 NFYC-AS1 NIPSNAP3A NKX3-2 NOG NOTCH4 NPIPA5 NPIPB3 NPIPB6 NPIPB8 NSG1 NT5C1B NT5C1B-RDH14 NUDT12 NUDT9P1 NUP43 NUS1 NUS1P3 OGFRP1 ONECUT1 OOEP OR2B4P OSGEPL1 OVCH1-AS1 P2RX4 PADI3 PARD6G-AS1 PASD1 PCNA PDPR PHOSPHO2 PIGW PINLYP PIR PLA2G2D PLA2G3 PLCZ1 PLEKHA1 PLG PLS1 PMCH PMS2P4 PNLIP POLR3E POU6F2 PPBPP2 PPP1R17 PRAMEF12 PRIM1 PRO1082 PROCR PRORSD1P PRPH PRR32 PRR7 PRR9 PRSS37 PTGES PTMAP1 PTRHD1 PTS PURB PVRL3 PWARSN PYHIN1 QRSL1 RAD51AP1 RASD1 RASGEF1B RBM20 RHEBL1 RHPN2 RLN1 RLN2 RNF157-AS1 RNF168 RNF214 ROR2 RORA RP11-1007O24.2 RP11-1017G21.5 RP11-102L12.2 RP11-108K3.2 RP11-10L12.4 RP11-1114A5.4 RP11-1260E13.2 RP11-164P12.3 RP11-16P6.1 RP11-190A12.8 RP11-248J18.2 RP11-250B2.6 RP11-27I1.6 RP11-307L14.1 RP11-307L14.2 RP11-319G9.3 RP11-365H22.2 RP11-384L8.1 RP11-400N9.1 RP11-401P9.4 RP11-438B23.2 RP11-445L13__B.3 RP11-466P24.7 RP11-471B22.2 RP11-485M7.3 RP11-510M2.2 RP11-513M16.7 RP11-550I24.2 RP11-568N6.1 RP11-588H23.3 RP11-669I1.1 RP11-67L3.4 RP11-722E23.2 RP11-753A21.1 RP11-945A11.2 RP1-239B22.5 RP1-274L7.1 RP3-336K20__B.2 RP3-388M5.9 RP3-428L16.2 RP4-575N6.5 RP4-621B10.8 RP4-695O20__B.10 RP4-798A10.7 RP4-813F11.4 RP6-201G10.2 RPL23AP22 RPL29P7 RPL34 RPL9 RPLP0 RPS17 RPS17P5 RPS26 RPS26P11 RPS3A RXFP2 RXRG S100A16 SALL4 SCG5 SCN10A SDCBP2-AS1 SEC24B-AS1 SEMA3E SGCE SGPP2 SH2D1A SH3D21 SHISA8 SHOX SIRPG SIRT4 SLAMF7 SLC10A1 SLC18A1 SLC23A1 SLC25A21-AS1 SLC28A1 SLC35G2 SLC38A11 SLC41A2 SLC44A3 SLCO6A1 SMAGP SMKR1 SND1-IT1 SNORA65 SNORD107 SNORD114-3 SNORD115-13 SNORD115-26 SNORD115-7 SNORD116-22 SNORD116-28 SNORD116-4 SNORD3A SNORD3B-1 SNORD3B-2 SNORD3C SNORD3D SNORD73A SNPH SOWAHC SOX7 SPAG5 SPARCL1 SPESP1 SPIN2A SPIN2B SPINK2 SPINK5 SPRNP1 SRRM5 SSSCA1-AS1 STAM-AS1 STX8 STXBP4 SUN3 SUPT3H SUZ12P SUZ12P1 SYN3 SYNGR4 SYNJ2BP SYNJ2BP-COX16 SYTL2 TAF1A-AS1 TARP TAS2R4 TAS2R45 TBATA TBX21 TC2N TCEAL1 TCRA TCR-alpha TCRDV2 TCRVA13 TCRVA15 TDG TDRD12 TEKT3 TEKT4P2 TGFBR3 THNSL1 TIAF1 TIGD2 TIGD6 TIGIT TLL2 TMCO2 TMEM108-AS1 TMEM117 TMEM192 TMEM220 TMEM231 TMEM61 TNIP3 TPO TPSB2 TRA TRAF1 TRAT1 TRAV12-2 TRAV12-3 TRAV13-2 TRAV16 TRAV17 TRAV22 TRAV5 TRAV8-1 TRBV5-2 TRD TRDC TRDV3 TRGC2 TRGV7 TRGV9 TRIM40 TRIM51 TRIM59 TSGA10 TSSK3 TTC16 TTC30A TUBA3FP UBE2Q2 UBXN7-AS1 UGDH ULBP1 UNC45B USP20 USP30-AS1 UTP14A VPREB1 VSIG1 WASIR1 WASIR2 WDR63 WIBG WNT1 WNT8A XCL1 XCL2 YLPM1 YME1L1 ZBTB1 ZBTB20-AS1 ZBTB33 ZC3HC1 ZCCHC18 ZDHHC11 ZDHHC11B ZFP30 ZGRF1 ZNF134 ZNF135 ZNF165 ZNF205-AS1 ZNF257 ZNF259P1 ZNF268 ZNF273 ZNF320 ZNF33B ZNF347 ZNF510 ZNF514 ZNF528 ZNF530 ZNF566 ZNF568 ZNF569 ZNF571 ZNF594 ZNF674 ZNF682 ZNF683 ZNF720 ZRANB2 ZSCAN12P1 |

**Table S4**. Gene symbol of 8313 DMGs

| **Direction** | **Gene symbol** |
| --- | --- |
| hyper-methylated (4636) | WNT3A VAC14 KCNJ12 SNX24 MAFG TFAP2A VTI1A THAP4 ZNF764 NID2 CREB1 PSRC1 LRRC47 LOC285954 HARS2 SMOC2 DPY19L1 DVL2 CORO2B KIAA1383 FBXL20 BEX2 ATXN3 GRB7 WASF1 NUAK2 SGSM1 TMEM139 KRT86 C4orf27 CNNM4 COL9A2 CYP27C1 LNX1 AKNA SGIP1 SYNJ2 C10orf107 SLITRK3 C6orf1 NAALADL2 SV2C HSPB11 FGF18 ALG8 DNAJC7 ZNF785 CLDN6 FAM109B CAMKK2 FUT10 TRIM47 MON1A THY1 C1orf123 LRRFIP1 LRWD1 C10orf58 ANKIB1 AKT2 LMO7 ATP6V0B SGK3 MPG ASB11 KLF5 LGALS2 HDAC4 SIPA1 TUBGCP4 SFRP1 ASB18 RAB34 EGFL8 MTHFSD ANKH KCTD3 UBASH3B HORMAD2 MS4A12 MLNR CDK4 CHTF18 NUDT4 RNF217 SMARCD3 HYMAI BBS9 ATXN7 ZNF718 XIRP2 LOC113230 LOC154822 LRDD PXK RCBTB1 MTL5 FAM49B DEFB136 EXTL2 TOR3A C7orf26 SPON1 GPAM KRT27 EVX2 MIR107 TNRC4 GNPTAB MYH3 CILP2 KCTD10 FABP6 PDLIM1 GOSR1 EFCAB2 C2orf65 TAF15 LCE1B LY6E DBNDD2 MTFMT OLFML3 ME3 PPP1R13B SCFD2 CNR2 PWWP2B PIK3R2 EDARADD MIR140 ICA1 AVP CTDP1 FCHO1 CPNE2 TBCD DAP NR2F6 IGF2AS FGFR1 CDK15 FLJ12825 MUM1 GDPD4 B3GNT8 BACH2 ACMSD TBX4 ZSCAN18 ARHGAP27 SAMD12 SRC TRAPPC6B HTR7P SHQ1 DPY19L3 PAG1 PITX1 H2AFY2 LHFPL5 SOHLH2 TNS4 TOX3 PLUNC NCAM1 COMP SAMD13 CHRNB1 WNT10B CSNK1D CRABP1 PRO1768 HECW2 PRRT1 PHACTR4 RNF207 KCNE3 ZNF18 SPCS3 KANK2 KRCC1 OPRD1 SNX9 MITF TMSL3 MARCH1 MARCH2 MARCH11 SEPT8 SEPT11 SEPT14 MAL2 PSMD1 ORC2L KIAA0895 NPTN NADK WNT8B QPRT ZNF462 C11orf9 MBD3 RHBG PPP1R3B BEND3 REEP4 DNA2 CAMK2D FOXJ1 COMMD3 DCHS1 LPIN1 DEM1 RIN1 AP1AR NLN SPATA17 KCNK10 GALNT2 MPP5 C18orf1 CXCL6 LEKR1 RXRA VSTM2B ARHGEF7 C3orf39 TBXA2R RANBP3L CLN8 EFTUD1 SNORA70B SNCAIP TCHH ARRB2 NKX2-1 RAC1 PDE6B NFIA TMX3 TIAM2 GDNF PRAC CLCN6 GJA5 GRLF1 ESPNL ERP27 DAD1L MAPRE1 LYPD5 AGPAT9 DYSF WDR86 SLC20A2 TPPP3 CCDC150 DDR1 RANBP17 SGPP1 FGR BIN2 ASB4 OTOR ZNF295 BRAF DUSP13 TMEM5 TM9SF3 MYPN C17orf56 INO80 TEF FOXC1 NEFH RPL6 C17orf96 IQGAP2 SEC24D RSPH3 MIR124-3 ERC1 PHTF1 PARN SLC9A9 GCLC ILDR1 CD2BP2 NTHL1 KCNA7 ACSS1 PSEN2 SPATA13 FOXN3 HNF1B ARID5B WWTR1 HOXB3 CTSK BCAS3 LEAP2 WNT7B SCGN LNPEP CD248 FAM65A ESYT1 EXOC5 LRRC66 FAM131C C1QL1 SLC22A18AS SYNGR1 LPIN3 TANC1 ASB13 SEC14L5 SMPD1 ZMYND15 GNG11 CEP55 MAPKAPK5 POMC ZFP42 CUL1 PTCH1 SIPA1L1 EMILIN2 PYGB ATP10A PRKCA MYO19 SYCP2L FBXO21 ATF7 KCNJ1 H2AFY DUSP6 GIGYF2 ELSPBP1 LIN7A KIF19 ZNF608 EEF1E1 ALCAM DEFA6 FAM190B GCA FGF7 RRN3P2 KIF5A TRPV4 ANG GMDS HEYL OXT DCP1B C3orf77 RNU5E EXT1 ACLY PPM1F LPAR1 STOX1 GPD1L SLC25A33 XPO7 VCAN MIR495 WDR12 GPR133 CCDC102B TRIB3 HSPB3 RPP25 HMGA2 RAI1 SLCO3A1 LOC100129034 HOPX TRAF7 MATR3 BAZ2A PTPN14 FLJ90757 NEUROD1 LOC285768 HLA-DOB SLC2A6 SQLE SLC6A16 REG4 OR4D2 SLFN12L SGK1 MYL12B ZMYND12 C22orf31 CLSTN2 ADORA2B FAM149B1 MOGAT3 TTC25 ATG4D MGAT5B TMEM45B PSD GALNT7 SORCS3 ELMO1 CHCHD6 SYNJ2BP CLASP2 TICAM1 C16orf87 AXIN1 SLC7A4 FOXK2 EPR1 TNR TUG1 GNAO1 TMPRSS3 CALR CNPY1 CACNB2 DKFZP434H168 FOXD2 MAGEC1 SNORD116-9 B4GALNT1 SLC25A44 KCNH5 PNKD UCN3 GRM8 FGF20 ABHD6 AGA LHX2 LRG1 TUFT1 ADCYAP1 PLD4 LTBP1 PTK7 POLR1E SIGLEC15 SOLH SALL2 IL6R INF2 FAM135A ARHGEF6 TSPAN5 RAB6B COL28A1 CSDE1 STARD9 C13orf31 MYO15B GOLSYN RBMXL3 PCDH9 MIR199A1 NFATC1 MIR374A SC4MOL NEURL3 TNFRSF19 LMX1B ENTPD3 TTC15 C11orf2 EMID2 CHST1 C1orf130 POM121C C20orf196 LY6G6F AP1G2 ADCY3 CCBL2 TDRD1 DHRS3 GALNT6 PPM1B FAM54B LOC100128811 TAS2R10 PIAS1 C1orf106 NHEJ1 C14orf93 OSTalpha NCRNA00167 TRIM9 GPT2 PM20D1 AP1M2 SYDE1 TXNL1 CARD6 NR2F2 GALNT10 MYCBP JARID2 CPNE9 FLJ32810 BCL2L15 SNCA SMPD3 GZMA GATAD1 HAL ARL8B TBC1D4 NCAM2 DDAH2 ZIC4 KITLG AGL DUSP3 WNT2 CCDC40 SFRP2 SELI LMCD1 VANGL2 PXDNL SLC2A12 DSTN TMEM64 NPHS2 RASGRF2 ZNRF4 MLLT1 GRIK1 GRM7 SLC16A11 SDCCAG8 MBP DUSP27 MRPS35 GFRA1 ZIC1 C16orf73 HOXB13 SLC35C1 BTBD9 ZFPM2 PACSIN1 MYH11 MBOAT1 TLR5 ZNF157 HPCAL4 PTPRU ANKRD5 SORT1 ASPHD2 SHC4 CCDC88B MGAT5 GDAP1 POMGNT1 C7orf38 CRH IGDCC4 EEF1A2 SRBD1 EMR1 GRIK2 MRPL13 TPD52 LRRC1 SPEF1 VENTXP1 HSD11B1 CCDC102A DZIP1L HLA-G RIN3 DLX6AS SLC12A9 BTAF1 SSTR3 ODF3B IMMP2L DNAJC12 C14orf182 TET1 CDKN2BAS ZBTB9 C9orf167 VWA5A KCNT2 HMG20A C17orf101 TTLL6 USO1 C20orf200 PRKCQ MED26 TM6SF2 CARHSP1 LOR ZSCAN5B PLEKHA5 EFEMP2 SALL1 CD151 LYRM4 ATP6V1C1 CSNK1G3 FAM113B EPHA2 PRAP1 MIMT1 CLC EPHX3 STK31 PAN3 RSPH6A FAM168A GPR110 DGKG MAP4K5 FAM5C C10orf128 VAX2 RIMKLB BIN1 KCNH1 DGKZ XPNPEP3 SERPINA12 FBXL17 PEAR1 LOC284837 ZNF391 SDR16C5 LOC647309 KLHDC9 FAM190A C21orf67 DTX3 SPAG6 RASL10B PRPH GLDC RNF10 KATNAL1 HMGXB4 MAP4K2 ADAP2 COL4A2 LASP1 FGF8 ATP6AP2 STX16 ARL10 C11orf53 TDRD12 RMST STMN2 CARD11 NPHS1 CLYBL OR2F1 MXD4 OR5AK2 IGFBP2 NCOR2 ARTN PPP3CA FEZF2 TEX13B ARAP3 SPINT2 RBM20 TRIP10 TUBA8 SOX17 SLIT2 INPP4A WDFY3 BCAP29 FGFBP3 MIR133A1 GPR172B LOC157627 ARPC5 SNX27 GSTCD PACSIN2 HCG22 PPT2 LTBP2 C1orf122 TRIM13 RALYL NR1H3 ABCB9 UTRN EPB41L4A S100A7A WNT10A TBC1D5 MYLK G0S2 MIR589 MIR1976 C1QL4 TMEM132C PDHB PCSK9 STARD4 MPP6 HSPBP1 CDK2AP1 FRMD5 GALNT3 PLCB3 SCARB2 NPM2 AACS LOC100130872 PRRX2 KIAA1267 ZSWIM6 IPO11 BMP4 LIG1 JAZF1 LOC100129354 PPP1R1B CLEC4A MKLN1 IL17RE KCNIP4 ZNRF2 KLHDC10 ATG2B PRRT2 PLCXD3 TMEM11 COX4NB DHDDS SLFN13 MYO3B RECQL5 FCN2 GNPDA1 DIAPH3 LOC344595 PICALM FAM18A C2orf69 SCUBE1 NUFIP1 SIAH3 S100B CDC42EP1 ACOXL DSC3 FREM2 SNORA60 ODF3L1 CCL24 YWHAZ SOX9 HS3ST2 MIR453 LRRC43 LTBR USP28 SPAG16 KIAA1804 RXFP1 GBX2 WFS1 SLC22A5 ESRRB MUC7 SYS1 SMTNL2 MIR639 NEU3 PDE4DIP TTC17 CAB39L CD302 CSNK2A1 NLRP12 GRSF1 TMEM38A CCDC148 PIBF1 GGA2 RUNX2 RAB15 YPEL2 DLG4 SCGBL LRRTM4 KRT38 SKAP1 NPBWR1 WNT9B LOC285550 COL4A5 SEMA6D AZI1 SEMA6B P4HB TMC1 KCNK15 TSEN34 KIAA1033 RBBP8 PCDHA6 PLXNA2 MRPL52 FLJ37453 SIM1 TRAPPC1 SLC48A1 TMEM56 POLR2A MIR635 KIAA0247 ATG9B ANKDD1A PAK1IP1 ESPN GTF2H4 TSPAN4 SYNC ALDH7A1 DNM1L CEACAM1 MED23 TGIF2 DCC VWA1 RTN2 ZNF717 ABCC11 A2BP1 TSSK3 CSF1 FAM124B TCF12 C1orf190 C12orf76 GJC2 TLX3 WDR8 SETD8 PSG5 WDR90 CCDC141 POLR2C C5orf62 ZNF490 ACACB PDXK RAB11FIP4 RPS19 FAM49A ALS2CR4 CLCN7 UBE3C SNORD115-17 PGBD1 PXDN OPRM1 RBM24 TRPM2 PEX7 CA9 RBBP9 FES SAMD7 LHX9 PCDH10 NDRG4 NDRG2 KLF7 WSB1 GULP1 LBX2 TGFB1 GUCY1A2 ZBTB8A RNF13 C5orf32 GPSM2 RGS10 LIN7B RGS8 MOG ZSCAN4 CPNE3 ZNF529 DLL4 MLC1 L3MBTL4 DLGAP3 PITPNA FOXA2 JUB GAL3ST3 GABRA6 RAD51L1 TPCN1 LIPE ICAM1 NCL SCG5 ZNHIT1 TGFBR1 RMND5A NOP58 IL1B DEPDC1B NKX2-5 C14orf105 FAM178B C1orf173 ZNF543 ZC3H13 CRIM1 DCAF17 PYCARD LOC254559 SLC17A7 LHCGR ERRFI1 MARCO HLA-DOA SAMD14 TPRKB TYSND1 GALNT13 TDRD10 MAP2 GIPR GNMT GALNT5 TBX5 SPINT3 WWP2 FAM169A KIAA1549 GPER C2orf3 RFTN2 SNCB VENTX DUSP18 DYNC1I1 C4orf43 CLDN1 BPHL RHOBTB2 HCN2 FLJ22536 BAI2 RPLP0 FBXO44 ANGPTL1 H2AFV GEM MYO1C BRCA2 UBTD2 DCTN4 TTL FAM138D SULF2 TMIGD2 TAAR3 SCARNA12 ROPN1L BMF PCNXL2 ZBTB34 MYO6 GOLT1A CDCP1 ZNF107 FAM149A STX1B PANK1 CDH1 ARSA LRRC56 WDR48 PTPN2 ATP8A2 SVIL PRH2 C1orf161 TMPRSS6 SLC6A18 ARMC2 C1orf204 TAGLN3 SPG7 HLA-DMB CPEB3 USP43 PPFIA3 TMTC3 DTWD2 PECI PREX2 NDUFA4L2 PFKL KRT1 TBL3 PPAP2C IDO2 JDP2 TTC21B IGFBP4 WNT2B TSPAN8 NEBL BMP7 HNRNPU HYAL2 RAPGEF3 HEATR7B2 ZNF609 LAYN FIGLA FBXW9 KRAS GFPT2 CLIC5 C1orf162 HVCN1 NEUROD2 MTA1 MIR620 ZNF714 CCDC85C ADARB1 PRLHR SMCR7L DERA C10orf26 ENGASE ZFYVE9 ERBB2IP KRTAP21-2 PDXDC1 C3AR1 NTSR1 TMEM68 RNASEH2B RORB PAICS KDM1A UNC93A PCDH11X CHRNA4 GLMN NEK3 SH2D4A KSR2 SCRIB TOLLIP MATK FREQ SNORA30 GP6 PURG DUSP11 RACGAP1P ZWILCH LHX1 ZNF660 PPP1R14A FAM193A CYB5A RGS22 PAX6 SNX22 PARVA CC2D2B TGFB2 CTBP1 SDR16C6 GRHL2 KCP LPPR3 PTPRR C16orf52 CHMP1A GPR1 UBE2E1 CDH8 VIPR2 PMP22 N6AMT2 FUT2 PTPRZ1 PPP1R12A PRDM13 LOC400043 TAS1R1 NMBR PDX1 RNF125 TRAM2 MIER1 PKNOX2 FAM71D GUCY1B3 TMEM26 FAM170A PI16 RGS7 TMEM99 CSPG5 PTK2 C4orf49 SLC6A19 LOC200726 UBA7 ZMAT4 PHTF2 MOBP HOXA4 TLR4 SSBP3 C22orf45 LOC221710 PFDN1 C17orf103 ZCWPW1 MTNR1A SCNN1A SYCE1 HEATR4 CCNI2 C17orf102 CLOCK TTLL11 KIAA1543 MOSC1 PGM3 OOEP CIDEA CD109 CTSA TNS3 CA13 CEP57 ZFP106 KIAA0495 WRNIP1 OPRK1 KRIT1 STXBP2 MARVELD1 NR2F1 SLC7A5 LOC100130933 TTC7A REPS1 CDH11 MMP11 FCN3 PDE6H ELAVL4 CRIP1 SESN1 WDR45L CTF1 ARSF LOC201651 SLN KRTAP10-3 TBX2 RHOB BOD1 NOTCH1 FUT4 GAPDHS FAM98B MIR130A TMEM108 CDK13 AMFR KIAA1409 ADNP2 CYR61 MIR206 SLC6A17 FARSA LMAN2L EPB41L1 CADPS NALCN ATP13A4 SLC17A9 LOC388588 LOC100129637 OR5D18 SPTBN1 FGF3 ASAP3 TCIRG1 TMTC1 C17orf53 CLDND2 COPS8 EPHB1 ADRA1D MORC4 PRELID2 ARID2 HMGB4 SYNE2 EPHA1 GSG1 NCR3 IL17A MEX3D SPOCK3 UNCX TMEM132D CRHBP MIR181D TFDP1 ITGBL1 MIR1256 RGNEF SP8 KLHL8 FRMPD2 GCLM APLP2 GCET2 MNAT1 ASAM SLC26A5 EMX1 TMEM163 SLC16A7 ARGFX PABPC1P2 SEPHS1 CYB561 SCN4B CCDC149 BEST3 ATP6V0E1 GATA2 GPR56 SNAI1 ECEL1 PPP1R3A OSR2 LRRC16A BPTF CNNM1 C3orf22 OR1L3 PLEKHO1 PEX16 SOX7 ZNF207 TSNAX-DISC1 MUPCDH MCOLN2 PRTN3 CD93 CCDC80 CCDC36 PF4V1 ANXA3 ESR1 TMCO3 SLC32A1 SNX32 ADORA3 SEMA4D PHYHIP MS4A5 LOC168474 RAB3IP NGFR SEZ6L2 NRSN1 UNC5D C5orf48 C7orf53 EFEMP1 RGL3 LILRA4 RAB42 C19orf33 RAB11FIP1 STX18 HOXB4 NOL8 APPL2 PDLIM3 PRSS12 CHST10 MIR10A NELL1 KCNA6 TSGA13 ZNF212 TBR1 TMEM132E UNC50 DAPK1 RBM16 NEDD9 C11orf85 ITGAE HSD17B11 ACTR3C DDX1 PAOX SHD DCAF5 BCL6 C10orf131 PLCG1 CACNA2D1 FBXO39 CLPTM1L RAB12 SLAIN1 YY2 SNRPN TGM5 TBC1D9B DTL THAP10 FARP1 ATAD2B KIAA1274 TBC1D1 ARHGAP24 EVX1 SOX18 STXBP5L ATL1 OPCML C16orf72 SREBF1 PID1 SSH1 C17orf91 TMEM200C MAP1LC3A PIK3C2B DSCAM OR4M1 WWOX KL BAZ2B CTAGE5 PLEKHM3 TAF2 C22orf30 DNM3 FAIM FAM82A2 IER5 TCEAL7 ANKS1A KPRP MTNR1B MACROD2 UHRF1BP1 DOCK10 WDR33 ATCAY MMP14 DENND2D TMEM51 APOBEC1 EEFSEC NLRP3 PHACTR3 IQSEC1 FHOD1 HAT1 HTR3C CPA6 PPARGC1A SMN2 LOC100302401 DPYS R3HDM2 TTBK2 LRRK2 TRIM27 PKD1 INTS6 MFSD6 CD14 PBX4 SSFA2 ZDHHC22 BEAN SRGAP1 BLCAP TEX11 LMAN2 MBD5 HEBP2 FLJ46111 ZNF354B PAX5 SUV420H1 RAB13 FLJ23834 AK5 KLK9 MAPK8 FLG GFPT1 OPN4 KIAA1522 CBLC HOXD8 LRRC33 THBS1 LOC401127 KRT7 LRP4 TMEM178 ASB14 GLRX2 ADCY9 LUC7L STK10 LSM6 C16orf55 LRRC36 SLC30A1 BTN1A1 NFATC2 TP53I13 ADCY7 SLC27A2 MSRB3 SNORA2B ARFGAP3 KCNB1 CHCHD4 C12orf43 CHN1 ISG20L2 TTYH1 RNASEH2A PPBPL1 DCTN2 SENP7 NEFL LBXCOR1 MAEL ZNF705G LTC4S DLX2 SF3B3 EFNA5 KLF2 HOXA13 NGF MEIS1 ATXN7L2 TRAK1 LOC100192378 FUT6 KCTD9 KLK13 TMEM176B MMRN1 ASPH IFITM5 JAKMIP3 GHR CPM SNORA38 CSNK1E PDLIM7 WDR26 SMARCAL1 UBE3A NSMCE2 BAIAP3 EEPD1 GLT1D1 TIE1 CCND1 LARP4B C11orf31 MBNL2 GYLTL1B TRIM17 UBA5 PLEK2 ZNF702P DPF3 ARHGEF3 LRRC2 FAM83A PAK1 PIK3CA GRHL1 LOC152024 SMURF2 MYH2 OR9G4 MFHAS1 MEIG1 HOXB2 LMO4 ESYT2 ANO4 ZFAND6 LOC646627 PHF20L1 VGLL3 NKX3-2 TUBGCP2 SPATA3 C11orf42 GIMAP7 TCEB2 RTEL1 KRT222 GPR6 ZNF696 INCENP PLEKHA1 ANKRD2 PPP1R10 ADCY8 C3P1 LRRC49 FZD10 FGF2 PRSS16 DEFB116 OR6B2 RFTN1 LCORL PSD3 GPC6 ULK2 AVPR1B RASIP1 C5orf51 C17orf71 SERTAD2 IL5RA ARL15 CBLB HYLS1 RTKN FLJ32063 KCNJ5 SCUBE2 CNOT4 RNF145 SPSB1 ZNF716 ATRIP PAQR6 RNF130 MIR1207 C5orf33 USP32 NFKBIZ DPY19L2P2 SNTG2 GSTM2 GPR180 SENP5 CD70 SH3RF1 KRT72 TSHZ3 TMEM135 TBX1 PHC2 VKORC1 COX19 DEFB123 MOCS1 HOXB5 ATP6V1A AGTRAP ZNF853 MGRN1 CDH15 LOC390858 ANXA13 FBLIM1 BRDT FBXO34 TTYH2 SYN3 MYH15 CCDC62 B4GALNT3 FKRP ITGB3BP MPZL2 SETD5 FBN1 TFAP2C P2RY1 COG2 MPO DUPD1 MYST2 GUCY1B2 TBC1D10A NUDT13 WDR13 NCK2 PDILT XCR1 RSPO4 ECT2L SLK RIBC1 DTHD1 IGFBP5 SLC8A2 GSTA4 KDELR3 PPIE RASSF8 AFAP1L1 NET1 FOSL2 KBTBD10 PFKFB3 CREB5 COLEC11 CN5H6.4 TNFAIP6 SLC18A1 APEX2 WDR88 TMEM87B HTA C6orf94 HNRNPUL1 BMPR1A DYTN CRISPLD2 HAUS6 CDKN1C GDF5 NXPH2 PSAPL1 MT1L C4orf32 PAWR CALB1 SMO MAGEL2 MAGEB6 SPIRE1 PDS5A PMS1 DHX37 PRSS45 RRAGD PARP15 C9orf98 MSRA FANCD2 SCAMP4 C2orf55 EVC2 PRKACA DRP2 C10orf95 LOC134466 ACCSL LPCAT2 ADSS GALR2 RESP18 ANKRD55 MAP3K3 SNTB1 LOC285830 FZD4 MINPP1 PTPN9 DAG1 CA10 C10orf105 KCTD5 PHACTR1 SNX25 MALL LEPREL1 UNC13C THSD7A FLJ44606 C10orf18 ZNF619 PDGFB COL14A1 KCNH2 ETNK2 DHRS13 BARHL1 ESRRG C10orf25 YTHDF2 FEZF1 LRIG3 CHGA ESRP2 EN1 KTELC1 FAM108C1 KLHL1 PIF1 VIP BTRC MLH3 UBE2E3 FBXO42 EPM2AIP1 KIAA1239 OR52M1 DMRT2 MSL3L2 CCDC53 TRAF3IP2 KDM4C SLC7A9 MNT HK3 RNF165 CAMK2N1 SNX31 STAC2 TBC1D2 TSNARE1 EXOC3 HLA-DRB1 RBM43 C6orf25 PLEK GLIS1 CD200 THBS3 ZNF652 BRAP RPS6KA3 MUC4 ESRP1 CTNNBIP1 CHD3 ZC3H12D ANKRD43 GLG1 MOGS PPP1CC AGAP2 TIMM8A CT45A6 FAM171B MFI2 UNG PTPN13 USP6NL TOMM22 ATP6V0E2 ITGB8 ENTPD6 MTM1 ENC1 TP53RK BCOR TRPS1 SRFBP1 PRKD1 C9orf30 C2orf28 DAZAP1 C10orf113 MIR222 IFT57 DNER CELA1 FSTL4 ATP5D FAM118A CATSPER4 PROC CAPN3 OR52K2 ZNF844 GJA3 FLJ10357 SIRT5 NFATC3 FBXO5 GRAMD1A C6orf141 ATP5A1 BRF2 TMEM159 KCNE2 LOC284661 TMEM151B ACO1 WDR70 NAT1 BTBD16 ZFHX4 AGPAT4 C7orf71 PHOX2B HRK PCLO FUZ GNA15 RCAN1 NCF2 PROM2 COQ3 NR4A3 LPAR3 OR11G2 SLC11A1 EMR3 CCDC115 YOD1 PDGFRA FBXO22 ATP2B2 P2RX7 CNTNAP5 MYCBP2 LGR4 BCR NR2E1 MTMR9L PICK1 WARS2 PLAC1 CACNA1E ANKLE2 CPT1A GFI1 C9orf4 CNTN5 PAX7 SLC6A5 TP63 C1orf69 TBC1D19 UNC13D RASGRF1 ANKRD39 PRDM8 VSIG8 NCRNA00200 HOXC11 TAL1 PLEKHA2 ARPP-21 FGGY VNN3 CADM1 EXOSC7 MAP3K9 PLCH1 NEK8 RNF135 SDC2 ROBO2 KLHL31 LOC100130581 TROVE2 FZD6 GRIA2 CCDC79 RILPL1 FBXL13 MS4A6E RASSF6 TPRA1 SLC5A11 APOM ATG12 RARA C13orf16 SEMA5A CALB2 ZNF239 ODZ3 PRMT8 GRB10 MAPK8IP2 SEMA5B MIR218-1 GMPPA HAP1 C11orf17 TP53INP2 C19orf54 SLC11A2 CWH43 CALHM3 POSTN CNST ABCC13 FOXL2 ZNF444 OXR1 AZU1 RHOQ SALL4 MAP1D LOC146880 ONECUT2 PNLDC1 UQCRC1 AKR1C3 SCRN1 LOC100190939 LRRC42 KRT20 AMN ZNF578 ABHD8 NEDD4L GPRIN2 PPFIBP1 MBD2 SLC16A1 LDB2 MAST2 LRCH1 COL9A1 EPHA5 LMX1A PGLYRP1 P11 TBPL2 CDK3 DHH EMILIN3 PADI2 HOXD3 BCAT1 TMEM100 CRYGD AURKC PRKG1 ALDH3B2 MMP20 POU6F2 PLEKHA7 SOCS6 RSRC1 IGLON5 KLHL5 LRRC3B KLC1 ACTA1 CCDC106 DNAJC5 MSRB2 C14orf68 ELK4 WDFY4 MEOX2 DHRS7 GPR98 PCK2 PRX SPNS2 MAP2K6 MADCAM1 MGC13005 THRA CENPN BEST1 SH2D3C PPP5C OR2L13 C20orf135 MIR199B BEX4 STMN1 FLT1 ITGB1BP3 LOC400804 GBA3 BBOX1 PRDM5 DLG1 CREM NAPA SFRS13B RSF1 EIF3L ZBTB16 SEZ6 C15orf41 C17orf104 PLA2G12A C7orf41 EHD4 ZNF521 C6orf162 GRIK3 CFH FGF9 CREB3L1 MIR127 HOXA1 INHBC FAM194B BBS5 NUBP1 RFX4 PDHX OR5K3 PER2 MEG3 GRID2 AQP5 FAM124A TINAGL1 ATP8B4 LOC148824 C9orf139 OTUD7B FLYWCH1 DYM PHOX2A BCAN SHROOM1 PDPN OSBPL8 DYNLT3 PALM3 SLC39A11 WNT5B CLCC1 XKR4 SLC16A8 SLC24A5 DUOX2 TRIL SLC36A2 C17orf55 TSPO2 SELE FLJ45983 FLVCR1 PTDSS1 PIGG KLB DNAJB13 GPR85 SMYD1 CKLF MYOM2 OTP LY6G6E WRB LMBR1 OR8S1 NIN P4HTM EMP2 VNN2 ZMYND10 PELI2 UBR7 EXO1 SERPINE2 TCHHL1 KIAA0564 FAM110A HELZ CRMP1 MAK16 QRSL1 ZGLP1 ALX4 REM1 DGCR2 ANKRD34C LOC645323 PACS1 HMGXB3 SUOX RBBP6 EMR4P KLHL34 LOC144571 LOC100128822 OR1L6 SLC2A4 SLC43A3 BRWD3 CNN3 C3orf26 SLC25A4 LOC440461 ACAP2 MAGEB16 NT5C2 FAM78A C4BPA PVT1 PTGER2 UEVLD MAML1 F2RL2 GPR158 ADAMTS19 PPP1R3C LARP1 WNT5A RFWD2 RAX LRP6 MAFA CD6 RELL1 ABR CHRND SLC19A3 ECHDC1 SLITRK4 MYO1B TSPYL4 ERICH1 TIMP4 ECSIT SDR42E1 PITX3 GABRB2 AGK ZMYM2 SYPL2 HTR7 QTRT1 OR6C68 PSMD6 PYROXD2 AGBL1 NOL10 PRR7 CYP19A1 ATP8B2 SGTB TGFBR2 C12orf68 FGF17 LGALS12 RSAD1 RHOXF1 SLC6A11 USP53 TLE3 C9orf50 QSOX2 BBS2 HOXD9 MIR609 GPR137C PDE2A PPP1R2P1 LOC654342 TBX18 PRRT3 CSRNP3 METTL2A C11orf75 BCL2L2 EIF2C1 PODN MMP23A LRPAP1 KIAA2018 SYTL2 ZFP28 FAHD1 SLCO1B3 FASTKD1 KRTAP5-7 ROR2 CDKL3 RAB26 C17orf76 OSBPL6 CDO1 HOXA2 PISD C18orf55 TANC2 WHAMML1 PIP5KL1 ICAM4 HNRNPF JAKMIP2 CHP2 NRD1 ATP2C2 NEK6 C6orf105 TCEA2 C1orf183 CD22 LOC286467 CXCL13 PDE4C ZNF514 WASL C14orf184 DUSP5P DLEU7 ACOX2 ZNF90 CDC42EP4 PLEKHA4 EIF3B C15orf39 OR9Q1 C9orf46 KCNS2 HPSE2 EMX2OS IRF7 KIAA0562 IL17RD NAA25 LRRFIP2 MAPK8IP3 RAB30 TUSC3 RASGEF1B SPRN RTN4R MIR141 SRD5A2 SLC6A14 ATN1 SLC30A9 TCF19 C21orf2 RAPGEF2 ZNF684 SPARCL1 TLX1 SORBS2 RCN2 LHFPL4 CRB3 AKT3 CHD1 MESTIT1 TTC33 NRGN NEDD4 SMTN OAS2 FAM57A BID DMD PRRG3 SRGAP3 FXYD5 ZBTB38 WIPF1 CALHM2 DST NDUFA6 KIAA1370 FCAMR ZHX3 CFDP1 PLEKHH1 CPNE8 CCDC33 CTNNB1 LOC145845 EFNB1 ARID1B CTNND2 FAM150B SNORD89 CHST9 C17orf63 GPR157 CHSY1 CYP11B2 ZNF136 KIF25 ACSL6 PEMT CRYBA2 MYOD1 IL10 GDI1 DDHD1 SOX5 WFDC12 MIR548N DNAJC17 ZNF621 CUEDC2 CEACAM20 FERMT1 IRAK3 LIMCH1 PXN PDPR GABRE TRIP4 ARHGAP26 CD247 TMEM184B MSH6 USP42 ELANE KLHL35 THAP1 KRT12 THBS4 MTMR15 RAB27A LGMN MYO5C USH2A OSTBETA CLIC6 TYMP RALGDS REST GLB1L2 MAML2 CACNG2 GHSR NARS STX7 GATA5 LHPP YWHAG EPC2 ARSB ITM2C ALOX5AP PCDHGA5 SNAR-D CXorf59 COQ9 OR1F1 NENF PABPC1 PTPN21 ZNF713 PMPCB RICH2 RBM11 FLJ44635 ZNF709 IGF2BP1 C12orf42 GRIN2A LOC100130522 LHFPL2 PLEKHA8 TUBGCP6 PTGDR SPATA24 KIAA0947 LDLR LANCL2 NCAPD3 SLC38A8 MIR196A2 FNDC5 MTMR7 ADCK2 WDR69 LOC285370 TK1 FLJ41350 MRPL32 ASTN2 HAND1 KCNAB2 AKAP5 IRX2 TYRO3 CHD6 ATP11C RCSD1 TCP11L1 RAB3IL1 TFEC DBX1 FAM89A AGAP11 SLC17A6 PHLPP1 LRRC37B ZNF454 MYST4 C7orf59 GNPNAT1 PDS5B TPH2 P2RX4 DOCK6 CAMK1G BRD1 TUSC5 PLCZ1 AP2M1 RALA C11orf88 PUM1 FAM198A SCAF1 CDC14B OTOS ZNF428 PQLC1 SETD4 RIPPLY2 TUBB2B SCGB1A1 NEDD1 COL8A2 HSD11B2 PRTFDC1 TNKS1BP1 CLEC4F LOC286002 RNF216 ZNF396 ZNFX1 NR1D1 ATHL1 LOC220594 CNTD1 RPN2 CLEC3B FICD GLA LRRC20 SLC5A5 NTNG2 NKD2 ZNF518B ZNF257 VSTM2A CFI BEGAIN LARP1B CAPZA2 LTV1 CYP2C18 NHEDC1 ADAR CHDH MYO1F TTC24 RELT ZNF267 PLEKHG7 GSTP1 ARSI RHOA MLL2 FLJ45244 SPTBN5 EP300 SCAMP5 FHAD1 RORC HPN CTCF ADCY1 ABLIM1 TSPAN3 BBX GPR44 EPCAM MSC TRAF3 SACS GP9 SLC38A2 IL22RA2 KANK3 FAM184A CHST14 KIAA1217 C20orf12 TOP1MT ERBB3 FBXW7 ADAMTSL5 AHSA1 SPINT1 TIMM8B TMEM185A MIR761 ZC3H12C SPG11 S100A9 DDX53 BAG3 RASL11B ERC2 CTPS KCNQ1DN CTNNA2 CETP SLC5A10 IRF2 EDN3 SPATS2 EIF4G3 APOBEC4 TGDS C19orf45 DNMBP RCAN2 MIR365-1 SNTG1 CXorf61 ACP2 C6orf115 AGBL2 PCID2 C19orf30 FGF11 UBE2MP1 DDX59 TGFB1I1 PGF RPS16 TXLNB ETV4 SEMA4C NKX6-2 AAMP H6PD AFF1 PENK GTF2F2 NFIL3 ART3 PTK2B MIR548A2 DOCK5 C6orf126 BCL9L AP2B1 FLJ42709 GLIS3 LIG3 PLD5 ZSCAN21 C21orf62 HOXD10 TYROBP SPEN HNMT TMEM62 WIPI2 BAD IGDCC3 ZNF860 MGP LIMK2 MIR196A1 CD59 RPL11 LOC731789 KRT9 GALNT11 TAS2R16 POLR2D TMEM201 C12orf49 HOXC13 PCYT1B TNIP1 GATA6 ZFR2 LAMA4 FAM162B MECOM PLAC2 FBXL22 VWA5B1 QRFP LAMB2L UNC80 ARHGEF15 PUM2 TGM4 KIRREL2 JAGN1 SLC7A8 PIWIL1 CLASP1 KIAA1614 CBX5 CLDN20 SYNRG CLU JAK2 CHMP2A ACOT6 GPR26 ADAM8 NSUN7 IHH HOMER3 RPS6KA5 RBM9 CEP72 GLCCI1 ZNF167 ATP10B MIR181C LARP4 OR4B1 KCNT1 MAP3K5 ASAP1 FRMD4B EIF4EBP2 ZNF738 C14orf143 GPR17 MAGEB3 CD300LG ZNF497 PKP2 ENPP3 ZNF471 KCNE1 GPX5 LRRN1 ROCK2 C9orf86 FBXO4 CCDC60 FREM3 SLC18A2 LCN6 GSG1L FBXO46 CNTN6 CCDC85A PDZRN4 LONRF1 LOC441208 ZNF536 MBOAT2 MAPKAPK2 ANO5 PALLD CRHR2 B3GAT2 NCSTN MICALL2 ALPK2 PHKG2 AOX2P COL21A1 DNAJB12 SLC10A2 MSX1 SNORD42B C20orf114 TNFSF11 SIRT4 ROBO3 RAB32 ADPRH OTOP2 CD177 BDH1 TECPR1 FAM19A5 FBXO25 OLIG2 EGFR LTF SUMF1 AMOTL2 LTBP4 PTPRM CCND3 ANK3 SIRPB2 EPHA4 ARHGEF4 KCTD15 LRP12 ASF1A SH3BP5 ASPHD1 PET112L LPAR2 LZTS2 ZNF664 NLRP8 LOC732275 LOC100132724 COL12A1 C8orf40 PTN NECAB2 SLC26A8 POLE ADAM11 FCRL6 GGTA1 TXNDC12 MUC16 KIAA1486 RNF208 LOC284798 EFR3A DTX1 ABRA ATF7IP FER1L5 FAM171A1 STC1 SSH3 LILRB5 ERBB4 HOXA9 PARD3 C17orf85 DLX4 HIATL1 IFNGR2 IL18 SLC6A7 SLC23A2 CXorf56 ABAT ZNF775 AVPR1A SLC44A5 HMBOX1 IKZF4 TM4SF19 SLC25A40 RAC2 HLA-H NOXO1 TGFA C17orf74 STIM2 SYTL3 KRTAP1-3 CDS2 HSPB6 CRYL1 C16orf5 NECAP1 ADD2 TGFB3 IL17RB CLIP1 HEG1 AMBN IRF5 VWA3B ITGA8 SNORD115-15 C1orf230 SNIP1 LMBR1L FAM189A2 SEPP1 PHYH SLC4A7 MAP2K3 GDPD2 LRRC6 PNMAL2 TMEM167A C22orf9 LHX8 DNHD1 L3MBTL C1orf92 MTCP1NB NKX2-8 A1CF RIMS4 RNF222 C3orf59 AMBRA1 TRIM4 PLEKHH3 NAPEPLD TRIM6-TRIM34 ELAVL1 HOOK3 ZFHX3 ZC3H14 PRDM10 OR3A2 B4GALNT4 LOH12CR1 MXRA5 PKP1 DIO3 FOXO1 POLR1B PRAGMIN TMEM134 IL19 GMPS PFKFB2 MYOT BRD3 NPY5R RAX2 ACVR1 FKBP7 PKD1L2 SLC25A36 TMCC1 TFB1M UBTD1 KRT16 B3GNT6 ENAH B4GALT6 TNFSF12-TNFSF13 IMPG1 RBM4B OPN3 MED17 PAH HAVCR2 WSCD2 CIITA STAR FBXO17 FUT8 PDPK1 ERGIC2 MED9 WDR20 CYP2U1 MIR514-1 CNTNAP2 MTUS1 ST7OT2 HIPK1 SIGLEC10 FAIM3 ST18 FLNB RPF1 BCL6B PLAU ALG14 GMPR ACSL1 ZNF667 ABHD15 GLS KDM5B OR4K2 MSR1 CPEB1 KDELR2 CADPS2 MAGI1 STRADB COPG ACYP2 SNX18 SPINK5L2 C1orf97 ARF3 CCDC160 ADAM12 OSBPL9 GRIN2C CCDC64 IL17RA LOC401463 KLHDC4 KCTD6 PDE4D PSME3 DHX8 SLC6A2 IPO13 PTPRA SP7 MMP9 CASP1 SLC35D1 ZNF509 DYNC1LI2 VHL BPESC1 KIF26B PCDHA1 C19orf75 SETD3 NFATC4 CD164L2 OAZ3 IL15 FBXW11 LYL1 ERI1 EFCAB4A LRRC4 RASSF3 ACRV1 CKAP5 GEFT EIF1AX FAHD2B GPR150 FXYD2 HADHA KIF20B ADD3 PRR24 SPEG FLI1 PRPF40A BTLA MRPL23 UNC5A TSPYL1 FAM63A NDUFS3 VSIG10L ARMC9 LHX5 WDR63 RNF115 GFOD1 LOC100128554 FLJ43860 NUDT16P PRDM2 SLC25A42 PTPLAD1 ATG5 IL17REL ABI3BP PLD2 UBE2O COL8A1 GOLM1 ANKRD33B GPR84 HSBP1L1 BDKRB2 MIA3 ACPL2 SMG6 ROBO4 DULLARD S100A8 OR8B8 OTUB2 TRABD NOV DYRK1A C20orf95 ALX3 RIPK4 VWF BTBD7 BZW1 ZBTB44 PHF1 IMPG2 ANKRD58 LRRC8D FAM198B FAM13C ATP5G2 POU2F3 GCNT7 TRPC5 EXPH5 TCP11 BARHL2 BOLA3 GPR142 TNIP3 CROT SMPX ZNF532 CXorf36 ZNF445 IL17RC C12orf56 ProSAPiP1 SLAMF8 GNG12 TPBG TPST1 LOC100131551 ZNF219 SNRPF MIR449B C9orf125 FAM84A PHF11 EYS FBN2 AMOT EFHB AURKA LRFN1 TBPL1 LRRC55 REM2 SLC25A10 HLA-DQA2 FAM48B1 TUBB MIR744 MGA WDR25 OXCT1 AKAP7 MAGEB18 RB1 SIL1 EDIL3 HSD17B4 TNFSF13B SCAPER DRD4 PCMTD2 PAM EGLN3 AKAP12 TUBGCP3 SPATA18 MMP27 HADH UAP1 NRP2 RANBP1 RSPO1 STARD6 UNC119B EXTL3 FBXL6 C3orf21 ZNF385D FAM186B NRSN2 NME3 DSG4 FLJ36000 SLC38A3 PEBP4 FN3K GSTO2 DEFB124 IFT81 HTRA4 AJAP1 JAK1 MPZL1 SPRED1 WBP2 DMKN XKR8 PDSS2 WNK4 RYBP EPB41L2 MAGED2 MXD3 POM121 MYO9A OIT3 RAB11FIP2 PLCG2 DLGAP2 RUNDC1 BOC TIPARP ZNF597 CASR LAIR2 HRASLS MIR128-2 ZNF800 ZNF17 AIF1 DDR2 ZC3H4 OVCH2 GPSM1 USP13 MIR103-1 LRTM2 RBKS MOXD2 GABRP ZZEF1 C17orf77 SNORA32 ABCC2 UBP1 GIT2 SSBP2 SCG2 PKD2L2 SPG21 WDFY1 CPAMD8 DLX3 LOC441204 HMX2 SFXN2 C9orf122 FAM123C PTHLH NNMT BTNL9 CHIC2 TKT PCBD1 LPHN2 CXorf21 MAP1A DAB2 RHOBTB3 FXYD7 NXF4 TBX21 DNASE1L1 MEP1B IFT122 SEC31B FAT1 GABRA3 OR5D16 NLRP7 KCNV1 CHRNA2 ANKRD28 BTNL8 USMG5 ESRRA PLA2R1 FAT4 SLC2A1 LRP1B C2orf68 VILL CATSPERB MIR212 RPSAP58 CASP10 ZNF415 KCTD1 C19orf47 TAAR2 EPB49 UBD ARVCF BZRAP1 ATP2B4 ZNF598 MMEL1 FZD9 C9orf130 GAMT POU2F1 C10orf137 KCTD4 SNORD116-5 HIST1H1A FAM114A1 XCL1 C1orf9 FBXO11 FIP1L1 PTEN DIAPH1 ALG13 PARP16 ELL2 INADL THYN1 FAF1 LOC286094 PDZD8 RUNX1T1 HEXA GPR135 HHATL NTN4 CDC73 PPP2R5A DIRC2 LOC151534 YLPM1 ACTN2 PLXND1 GPD2 TMEM30B TTC23L PDZD4 AP4B1 MRPS6 LRRC18 GPR160 ZNF704 POGZ SLC25A26 KIAA1598 SKINTL TCEA3 POLR3C VDAC1 PAX9 ZNF91 ATRN FKBP9 RYR3 DAB2IP TSPYL5 FAM78B LRP2 ZNF793 TSLP TRPC1 ARHGAP25 TMEM74 POU2F2 HSDL2 HK2 HPCA OR5L1 PDE4B NKX3-1 FNIP2 GPR12 SPATA5L1 NAA30 CDH6 LDLRAD2 GPX3 MIR148A MYO1A NUAK1 GRM5 C8orf45 CEP68 APBB2 EPS8L1 PRKX METRNL MCC COX7A2 ZNF337 FHL1 ITCH FOXO3 PRELP RNASE7 ZNF124 SP5 MACC1 KRTAP4-3 ADAMTS16 ZNF862 OSCAR TOM1L2 ZNF138 MFAP4 LOC146481 RALB C19orf51 KBTBD11 PNPLA3 USP10 CHKA RMND1 RSU1 ANGPT2 ATG7 USP22 TMEM19 SLC6A1 LOC149837 IGFBP3 KIAA0664 GABRG3 TMPRSS11A GABRA4 CTDSP2 FAM134A UBE2C SLC29A3 MAP3K6 NEIL3 JTB C19orf62 EN2 SERPINA1 ZSCAN1 C6orf127 ISL2 TRERF1 HOXD11 FGFR2 APOL1 RAB37 MT3 FKBP5 VPS37D C6orf146 PARVG SNX8 SEMA3G DNAH6 GDF10 ANGEL1 MIR7-3 SMARCC1 PRR5L SSR2 C14orf162 AREG PFKP LCA5 IDS SLC5A12 TSC22D1 FAM13A OR5M1 C1QTNF6 FAM59A GTF2IRD1 C2orf42 SLC25A11 SLC13A3 SPATA5 SNORD116-11 LHFPL3 ZNF311 HSPA4 IFITM3 KIAA0748 ZXDC DOC2A PSMD5 PHLDA3 DDX4 IGF2R KCTD14 KIF5C VPS24 MSTO2P ULBP1 PAK2 FOXJ3 CBLN2 PROKR1 UMODL1 E2F7 KCNAB1 NONO TLX2 CTRL RAB22A ARHGAP20 RGS9BP UGT8 OR4F6 ZNF283 KIF22 HCG27 RNF20 LOC100133469 SHB LRIT2 ZNF165 IGSF3 ZNF263 FLT3 KRTAP2-1 SLC6A15 CAPN7 SERPINH1 ABCA13 ARHGAP18 CCDC109A EIF2B2 GHRLOS FAM65B TMEM8B KHNYN LETM2 PRPS1 HMGCLL1 PARP11 ERCC3 NPR2 COBL GFI1B SEL1L3 MYO10 ADAMTS5 CCDC9 SNX16 ZHX2 LOC283856 MEX3A FLJ43390 FAM59B LRRC28 NEK10 MTMR2 MXRA8 MICAL3 LSM10 ALOX12P2 C1orf174 FAM26E PDZK1IP1 MMD RP9P IRS1 AGXT2L1 OCIAD1 FAM55A TMEM93 EGLN1 CASP9 UCP1 LGALS13 ZP4 RNF128 C3orf57 SNRPD3 BCL7C MS4A13 HSD17B12 VAV1 NFATC2IP SFT2D1 SRGAP2 KIAA0922 MMP19 NCEH1 MIR1278 PORCN ZNF710 HSF5 TPO ESM1 WDR44 KLK12 C2orf79 TSC22D3 DRD3 C15orf32 FGF12 ABL1 MYBL2 VCL WIT1 PPAT HMGA1 TMPRSS12 RBM19 PDZD2 USP50 PPP1R3D TSSK6 GPR149 C1orf70 LIN54 SEPT7 SEPT9 SEPT10 FAM163A IGSF21 MAGI2 MTSS1 TRIM26 HOXC4 ODF3L2 TNRC6C DEDD PALM NFIC PTPRG PTPRN2 ODZ2 ST5 KCNQ1OT1 LYST SPOCD1 AFAP1 DLG2 PRKAR1B DOCK9 RUNX3 GRAMD1B SYNGAP1 MUC2 MAP4 KIAA0319L HCCA2 KIAA0513 SAPS2 ACHE COL9A3 IGF1R MACF1 HRH3 CD81 CD3G FOXP1 PIP5K1C HOXA3 APITD1 RAPGEF6 TNXB TIMP2 MAPK15 RERE NFAM1 VPS13D TNRC18 ANO1 RPTOR P2RY2 MYT1L KDM2B NEB BAHCC1 ATXN7L1 ETS1 FCGRT PHF21A ADARB2 CENPP TBC1D16 PRDM16 SLC12A7 TLE2 SND1 AP2A2 GRK5 ZFPM1 MAD1L1 COL5A1 TNIK SLC13A5 BRUNOL5 NRXN1 ARRB1 CCDC55 RNPC3 GALNTL6 PLEC1 BAT2 ZNF238 GRAMD2 ZDHHC14 NLGN1 DHX16 SLC9A8 JOSD1 AGAP1 EXOC2 KCNQ1 CACNA1C ARHGAP10 GNAS ZC3HAV1L TJAP1 ANKRD11 C15orf33 AHDC1 HOXA10 RNF39 FAM131B DPCR1 UST MYST1 RBMS1 NF1 COL22A1 GNA12 MBOAT7 CCDC92 OSBPL5 CADM2 MRPS16 SPATS2L UNC93B1 TTC7B ACOT7 SBNO2 NR3C1 MTUS2 GAL3ST2 TP73 MIR548H4 ARID3A MXI1 ZAP70 CAMTA1 RBM47 ARIH1 MGMT MGC34034 FAM101A LOC404266 KCNMA1 PITX2 C1orf86 ACACA KDM4B PTRH2 B3GNTL1 GLP2R SKI MUC5B BCL3 PCGF3 CENPL SGEF CNTN2 ZNF420 ARHGEF17 C19orf38 SHANK2 NHEDC2 SLC9A3 EVI5L LSM4 NCALD PLEKHN1 ZNF423 KNCN PRR3 CLK4 SMCP HIVEP3 GATA4 TTYH3 CUX1 THOP1 LAMA2 JPH3 CLEC11A DMRTB1 RING1 BTNL2 BAIAP2 LARS2 WDR60 MCF2L2 IREB2 TEX14 RASAL2 DNMT3A MEST TCF7L2 RASA3 NIPSNAP3B TNK1 RBPMS KLHL29 FAM172A DPYD TTC18 CDK6 IFT52 WBSCR16 CBX2 SDC3 EXD3 LAD1 MARS2 NAV1 CBFA2T3 OTOP3 RTN4 NOTCH3 NFIX DYNLT1 MAML3 MTG1 RPL41 CALY HS3ST3B1 SLC12A4 KLC2 NOS3 GNG2 PHC1 LOC283999 DIP2C BCL11B DOCK1 MIIP HEATR2 C7orf50 GBX1 STK39 SGTA KIAA1688 ITGB5 ATPGD1 SLC44A2 FAM102A NUMA1 C1orf51 MAP4K4 PPM1L NBEA RGS12 LOC100130987 PRKCH HOXB7 CCDC108 PRKCZ ERI3 PRPF8 CLIC1 COL4A1 PNPLA2 AGER SLC22A11 SOX2OT RGL2 PELI3 HMGN3 ANXA6 EPHX1 KCNQ2 KIAA1751 ZBTB12 KIAA0182 DTNBP1 AHRR MAEA BZW2 RIMBP2 CLUAP1 PEX14 NADSYN1 TIGD3 SPI1 GALNS GPR4 VGLL4 IQCE CCDC46 TRIO CAMK2B KIAA1026 DOCK2 CCNDBP1 TNNI2 ECHDC3 NOTCH4 PPP2R2B C1orf101 NCRNA00171 HS3ST1 NES CALD1 MYH10 MYOCD C8orf73 SLC22A18 RREB1 ATP11A LRP5 ARMC7 GABBR1 RADIL GRIN2D MDFI ARFIP1 SLC38A1 C6orf106 GALNT9 SFRS8 ZBTB41 UNC5B PITPNM2 IGSF22 DSCAML1 PARD6A FRMD4A CCDC88C TERT FLOT1 DLC1 SETD2 CHD5 RASSF5 SLC16A5 FAM176B TRAF3IP1 KLHL26 NAV2 FXYD1 MAP2K1 ACSL3 PTPN23 RILPL2 ADAMTS2 VSX2 JAK3 MAN1C1 DAXX SEC61A1 RPS6KA2 ZNF10 ORMDL2 LBH AKAP13 PDLIM4 ZNF148 CYP11A1 FBXL7 ACSF3 STK32C HAPLN4 CSMD1 KCNQ4 ANKS1B ATP6V0A1 TMEM89 SSX2IP COMT FAM115A KIF7 EDAR FAM155A KLF3 WDR46 AMPD2 LY6G5C RAB7A LMNA SOX1 GMEB2 ATP9A AHNAK PCDHGA4 STAB1 CACNA1H IFFO1 HCG9 GFAP DDX54 PC SLC16A3 TSPAN14 SDK1 CARD14 PRKAG2 RBMS3 TULP4 HERC1 RAP1GAP2 PLVAP ANGPT1 EMP3 FAM38A SLC44A4 PITRM1 CS RAB1B OCA2 ETV6 CTSZ PARD3B KPNA2 SLC38A10 C6orf89 KCNK3 PHACTR2 LASS4 EIF4G1 FBRSL1 SLC9A1 GRIN1 KIF1C NPR3 SLC5A9 SHC2 CDC42BPB MTF1 TNPO1 EP400 TPPP TNS1 RNH1 GNG13 SLC6A3 INPP5A ANKFY1 MRC2 KCNJ10 TAP2 INO80D C9orf171 FNDC3B C9orf25 SNED1 C1orf159 GRK4 PPARA KIAA1324 KIFC1 SCARA5 KCNA3 MAST1 NBLA00301 DIRAS1 PGCP DOCK4 MEIS2 LRRK1 C1orf175 MAPK10 SLC22A17 PLEKHB1 C19orf35 MKX TRPM5 CHPF2 OSBP2 ANK2 EYA4 LENG8 PEG3 SLIT3 SHANK3 ZMIZ1 C6orf48 MMP15 TRIM2 AMOTL1 COL5A3 CALCOCO2 C9orf173 SEC22A HRNBP3 NECAB1 COL6A2 BANP MEOX1 CGN DGKH IL12RB2 FHL3 PRSS33 KIAA0284 TRIM40 SHANK1 FZD5 ACCN1 AMPH HCRTR1 C8orf33 COMMD2 SYT7 PER1 IGSF9 MYO9B LYSMD2 TGFBR3 SHISA9 COIL TMC8 ZNF644 ATF6B EHMT2 FAM107B UQCR SEMA3A PHYHD1 EVL ZBTB7A RAB8B CAPN5 C5orf4 C21orf29 COL1A1 POFUT2 COL11A2 B3GAT1 DNASE1L2 ZNF783 HOXC9 ARHGEF10 B3GNT3 CAPN9 PPP1R9A RPAP1 COL6A3 BRUNOL4 FAM131A GPR153 SLC12A5 CD44 F10 IL28RA MAP7 SGCE MAP3K8 SOX6 NDUFS2 CREBBP RNF220 SRRM4 ITGB2 ASPG RAMP1 OTX2OS1 PPP1R3E KCNJ6 LRRC32 PELI1 ST3GAL4 SH3RF3 AP2S1 ITPKB TRIM31 TEAD1 PLK5P FOXK1 TCERG1L SEC14L1 KCND3 C21orf33 CACNA1I TNFRSF8 CNGB1 LMF1 NPHP4 HERPUD2 PLIN1 POU6F1 ADAMTS13 TBC1D14 ZC3H12A IKZF5 AHR UGP2 HLA-DMA SLC4A2 TRIM39 TCTE1 C5orf42 NDUFS8 PRLR RRAS2 GNG7 PACRG FANK1 CACNA1A DUSP10 LTB SLC30A7 DOK3 RFESD ZC3H18 MAPK8IP1 PCDHA2 CCDC67 AGAP3 C7orf20 KNDC1 HLA-J SLC22A6 LSP1 C10orf82 CLEC4C AGPAT1 SORCS2 ADCY4 TMEM183A MFSD7 CXXC5 CTBP2 NEUROG1 RASGEF1A GNL1 TRAF6 DYNC1H1 RORA DENND4A FNIP1 P2RX1 CRTAC1 PHLDA2 ZBTB22 FGF19 PLCH2 THAP2 AGPAT2 OTUB1 GRID1 HLA-DRA MDGA1 PLAT CLSTN3 FGD6 GNASAS CACNA2D3 DGKE HIF3A FAM125B HIPK4 SRRM3 GLI3 S100A16 ZBTB2 ITPKA OGDH NRM SPTBN4 PAX8 IQSEC3 CDK14 MEGF8 C20orf117 JMJD6 FBXL18 HLA-F VARS2 NXN EIF4E1B SLC9A3R2 FNDC3A EIF2C2 AATF RYR1 SETD7 CTTN TATDN1 STAU2 RASGRP1 FHDC1 IFT140 CHST11 ASCL2 PCDH21 NTM SEMA4B FAAH TMEM217 SHC1 PTRF PGD ZC3H3 TRPC7 AXIN2 ST7L BAT3 SCOC ANO6 ADAMTS10 STARD3 RPH3AL C10orf71 ADSSL1 NTRK3 KIF13A ADAP1 POLR1C STEAP2 C6orf114 COL7A1 CCBE1 FCGBP VEPH1 ANK1 EGR3 PSORS1C1 METTL9 RFX1 XKR6 GNG4 USP36 NFKB1 SLC1A2 SLC22A3 PDE4A MYBPC2 FTL PSMA1 NRP1 SIDT1 LPCAT1 CARS2 C5orf13 RHOBTB1 C3orf50 COL18A1 WIPF3 ZNF763 SFXN5 BAT1 BTBD12 ELF1 ARRDC2 TIA1 CLSTN1 ZNF556 AGBL3 RINL FAM19A2 SCHIP1 PMEPA1 CCDC85B KIAA1462 AAK1 TRAPPC9 ZNF365 BRE TSPAN9 SEMA6C LLGL2 PPAP2A MCF2L CHL1 CDC14A PRSS22 ULK1 POLG FASTK RPS9 KCNH3 GTDC1 ZNF469 STK24 MYRIP MZF1 SNORD116-17 MSI2 SLC8A3 COL6A6 CDKN1A RFX7 KPNA6 MLPH GAK RICTOR TNNT3 PIGV AHCY VAV2 DBP CHRNB2 NXPH1 MACROD1 TH FAM20C BRF1 TTF2 DOT1L OBSL1 MIS12 ASPRV1 FGF14 LRIG1 NIPAL3 GRIN2B MAPK12 TRIM15 HNRNPM ZNF354A CASP3 SSU72 HIC1 MOV10L1 TNK2 SLC39A4 BRD4 RGL1 SLITRK5 CHID1 ARF4 ALDH3B1 ZNF614 ENPP6 C10orf53 CCDC48 EFCAB6 RAB3C CDYL SUCLG2 NRXN2 SCN4A SCARF1 ACTN3 C9orf68 PTTG1IP CUX2 MEFV LRP1 MYOZ3 KIF3C CD300C OS9 LOC339524 CYFIP1 KCNB2 SHF PLEKHO2 AUTS2 PTPRE SLC2A5 SUCNR1 ATP4A GRM2 PAK6 CDC42BPA BMPR1B PCSK6 CAPZB INPP5D C10orf96 NOL4 P4HA1 SCD PLXNA3 NR5A1 UNKL MSLNL RALGPS1 C10orf90 UNC13A KIAA0556 EPB41 GRIA1 VTI1B NKTR CAMKK1 CRHR1 PLEKHG4B WWC1 C14orf181 PLA2G2F S100A11 ZCCHC14 KSR1 ADAMTS17 LRRC27 C22orf33 CDK18 CDH13 MTHFD1L RASGEF1C ZFC3H1 CACNG8 GRM4 NHSL2 HIPK2 DEGS2 LPGAT1 CMAS SSTR4 CALN1 LEMD1 ZNF565 NHLRC4 CMIP DNAJB6 TSKU KCNS1 PLXNA1 APOC3 NKX6-3 PHLDB1 DDC ODZ4 C6orf108 SSC5D RHBDL3 SULT1A1 FMNL3 C3orf64 SH2D3A AATK SEMA4A XIRP1 PTK6 CAV1 KLHL6 PIGQ C11orf41 BNIP3 MST1R LGR6 MPRIP ISYNA1 HOXC12 EZR WDR27 CLPS ZNF706 RAPSN ASCL4 FLYWCH2 GGPS1 SETBP1 ZNF827 RDH12 INPP5B IL27RA TBX15 MED24 DIP2B CCDC78 IQCG HDLBP CECR6 CFD LGALS3 CASZ1 GPR176 DLX5 IFNGR1 IKZF3 KDM6B PAPLN UAP1L1 SCAND3 DPP6 GDF1 ZNF385A GRIK5 SLC12A8 TRPM4 WDR37 DUSP16 GOLIM4 FILIP1 RUSC1 C12orf72 CAPN2 CTLA4 SAMD4A CBX4 ABCG8 SLC35F1 SEC22B JSRP1 ISLR ANKFN1 SCNN1D IMMT CRADD LDB1 PIP5K1A RPS15AP10 TRRAP ECE1 ARID1A SH3PXD2A DENND2A MARK2 TDH UVRAG GPSM3 KCTD11 P2RX5 MYADML2 PLEKHA6 HMCN1 RHBDF2 SOX13 ST6GAL2 TET3 SLC4A11 VPS53 WHSC1 SLITRK1 TMEM131 ZEB2 GNS GALNTL4 SLC7A14 SPATA20 DNAJC8 ZBED5 NODAL SRRM2 TFAP2D ADORA1 PLXNA4 DOK7 MKS1 STRN MAP1B UBE2W SH3BP4 HSD17B13 VPS13B ERBB2 SRCIN1 EFNA2 ABLIM2 DHRS7B BICD1 VGLL2 VPS45 DRD2 LTB4R SCRT1 MGEA5 CNOT6L PTDSS2 PIGR PHF21B ONECUT3 ENO1 SLC15A1 CHRM2 C6orf176 EFNB2 FAM45B RNF182 LPAR5 ROR1 STK19 CYFIP2 BTBD11 KCNIP2 PCNT CPA5 POLQ LY6G6C AFF3 MRPS18B MED13L LIM2 CDH22 TXLNA ARHGEF10L SETX ABTB2 C21orf57 PNPO SPNS1 TMEM167B SPOCK2 TEAD3 ANGPT4 C15orf52 C6orf10 ADAM32 PLXNB2 S100PBP RBPMS2 CATSPER1 KCNJ9 PPP2R5C C20orf24 TTC26 ZNF331 AKAP8L MIPEP STXBP5 COBLL1 ABHD2 WTIP FXR2 CXCR4 USH1C PLXDC1 LOC100133545 HPS4 ZFYVE28 SDF4 LOC283663 NEU1 PPME1 SCTR PAFAH2 SLC45A1 NRG1 |
| hypo-methylated  (3677) | RPAP3 IKBKB TRIM10 STOX2 B4GALT3 SLFN5 BCL2 CUTA ATP5G1 TCF25 CEP97 PACS2 MTA3 KIF3B ATM MYO18A LOC646498 PASK SPCS2 DFFA NCRNA00188 LTA SFRS1 FAM53C MKL1 TNFSF9 TMEM205 VPS52 NXPH3 PLK3 UBE2D3 HSN2 DTYMK CDC23 ZMYM5 GSTZ1 LIMK1 HIST2H2AB IL10RB ALOXE3 FUT11 HDAC5 PXMP4 MAP3K12 CASP8 MGC70857 MYH9 SSTR5 MTMR14 PRKAR2A ZNF211 CLCF1 IGFL2 CD36 MED16 RPL38 POLR3K ATOH7 PSMB9 KIAA1949 ATP13A1 CRYM HIST1H1D SMARCE1 LRSAM1 PTMS NAT10 CALCR EHBP1L1 MARCH2 SEPT1 SEPT2 SEPT4 PLEKHJ1 BEST2 ZNF829 NEAT1 PILRB ODC1 COQ4 SEC14L4 FAM83D NR6A1 SLC1A5 NCK1 MAPK1 CNOT2 CHUK MCM6 MB NAT15 SGCB TAPBP PTPRC TRIP13 USP47 ITSN1 SMARCA4 LOC400931 BSCL2 DPPA3 SIRT7 DYDC2 RPS3A FAM71F1 MTBP SLC47A1 ZNF560 MSH5 TMEM107 FLT4 ORC1L PGS1 KHDC1L NAB2 CARS MAZ VSTM2L FLJ26850 RWDD2B VPS37B C11orf87 SIN3A MAPT NUP85 SATB1 KCTD2 ZBTB1 C1QC ZC3HC1 GPR125 ZNF574 KIAA1147 OR4D11 PDCD1 TMEM219 CDKN2C IFIT5 LRRC41 PCYT1A CASC3 IPO4 SRRM1 HMGCL HSD17B8 IQCD TAX1BP1 PSMD3 CBFB LTB4R2 HIST1H2AC CSMD3 CHRM4 PLA2G4C ATP5L FAM69C PCP4L1 SLC35E4 LOC285419 TMEM145 ITGAL HMHA1 RNF8 SBF1 LRPPRC SAMD4B GNAT1 CLK1 SEC14L2 PAN2 PRR14 FOXO4 RPLP2 OLFM1 KIAA0895L DDX21 MEA1 DMBT1 EML2 CDC7 IRF8 B3GALT4 MEF2D ARL16 TESSP1 BCAS4 TMEM88B DGKA PRDM14 MIR483 NDUFS7 KAT5 LOC145814 SACM1L NUPR1 CCHCR1 HIST1H4E CACNG1 BAT4 H1FOO TMEM66 LOC219347 NDC80 LOC100128977 PML ZNF350 RNF4 BCKDK SLC2A4RG KLHL28 C3orf32 SDCCAG1 COX5A MRPL16 UACA ZNF880 EIF1AD RPA3 HYI ENSA C20orf199 PTCHD3 C10orf79 BTBD2 ZNF230 SERPINB8 ZNF132 STX12 UNC45A SLC1A7 DNAH17 NYNRIN DYNLL1 RTN4RL1 RPS15 JAM2 PTOV1 CLDN14 CDK12 EPN1 UTP3 RTN1 ASXL2 EHMT1 RHPN1 MSL2 NETO2 ADNP PDE9A CRTC1 GAD1 FAM22F CACNA1G PGAP2 SF3A1 SYK DNAL4 KCNC4 PEX11B IGHMBP2 ACTC1 TPSD1 DDX17 ARHGAP23 RECQL TP53BP1 CYTH1 ADHFE1 SP2 HSF2BP FAM83C KIAA1530 KIF6 AKT1 UBAC2 SIX4 UBE2B TECPR2 CCDC94 HECW1 UCRC MS4A10 MSLN POLR2E RSL1D1 MIR1185-2 PFN1 TBCC EIF3H SRF LENG1 PHF12 NAPSB TCF7 MICB SEMA3F SNORD24 METTL3 C17orf68 CYB5R1 AP4S1 LBP MIER3 TMCO1 LOC643406 CENPM C22orf23 RAGE FAR2 ZNF547 TIPIN EPPK1 COL23A1 RBM8A TMBIM6 TAP1 LPCAT3 IL1F5 CAPZA1 MGAT3 STX3 C1orf127 EDNRB SEC1 HSPA1B ANKRD42 AKNAD1 GNL3 BRD2 CD4 HLA-DPB2 LFNG HIVEP1 PDIA2 ZNF224 COL20A1 IPP PIK3CD ZNF776 RBBP4 BNIP3L IKZF1 KCNN4 LOC619207 MED20 POLR2G GPRC5B NLRC3 MIR1178 TUBA1A HHLA3 OR52B2 SEC11A NDUFB10 KIAA1737 ZNF564 E2F3 CCDC114 C1orf177 NANP ZC3H11A MAST3 KRT25 OBSCN CATSPERG PPP3CB GTPBP3 SEC14L3 CCNL1 KLHL21 PIGT HGS GMEB1 KALRN PCDHB5 KCTD18 MGC23284 HECA IL17F PTPN7 TAF11 ARHGAP22 FAU SFRS5 SLC12A3 SNRNP25 RGS20 STK11 NDUFA11 MCL1 MORN1 MCM7 CORO1B TRIM54 AMZ2 PUS1 DPYSL4 TAS1R3 UGGT2 BDNF ZNF581 IGSF9B SLC46A1 OR10J3 DNAH10 TOP3A SNHG12 KRT18 HNF1A MCM8 LIN37 HEATR1 UTS2 GPX4 ZNF260 TNF ABCA2 HEATR6 NACA CDSN TPSG1 TMEM59 DCTPP1 NUFIP2 ATF2 MLL RAI14 HIST1H2BK DAGLA COASY CNN2 PNKP CD300LF MED1 BLOC1S2 ZSWIM5 GPHA2 GABPB2 FSIP1 LPHN1 NUP62 UBE2Z GGA3 GPT ARHGEF1 DNMT1 GNB1 HLTF EFCAB5 IFIT2 PSMD4 PPP1R15A MIR150 NR2C2AP RAPGEF1 FSCN3 PIGF EPHA10 TMEM126A GPR113 ANKRD35 C6orf47 EYA3 POU4F3 TTLL8 AP1S2 PDE6D KIFC3 ARRDC5 MAP1LC3B2 CDX4 PPFIA4 SLC2A11 PXT1 RAET1K HSPG2 HACL1 SLC34A1 IFITM1 HNRNPC PA2G4 ERCC5 RHOD HIST1H4L ALKBH5 TXNDC11 COPZ1 NAP1L4 ANAPC7 ZNF649 GBP4 ZNF749 DRD1 CIZ1 KIAA0195 MYST3 FAM109A MICA GABRA5 DCAF8 ACR TMCO4 CYB5B KIAA0892 KLC4 NCRNA00111 FBRS SERPINB9 TSPAN32 APEH MAFF PSG9 ZNF141 HIST1H4A UCN2 TMPRSS4 CADM3 ITGB4 SFT2D3 C12orf57 BAT5 SNW1 ITGA2B ICK ZNF557 ZNF570 ARPC4 TNFRSF4 FKBPL SLC34A3 APOB PSMB7 TIAL1 AIMP2 OSM DHX34 USP15 C10orf76 ZDHHC5 TG FBN3 EDEM2 ACOX1 NOS1 WDR53 CYP11B1 RNF183 PRMT1 KIF15 PRKCG EPB42 MLLT6 C20orf71 ELAVL3 KCNE4 CA12 GNB2 CELSR1 TMEM80 KCNJ4 ABCF2 DNAJC28 RPL18AP3 CWF19L1 ISY1 JMJD8 ADAM33 TAF7 SIPA1L2 GAS7 SFRP5 INTS2 MRPS7 RHOU TMEM150C LMNB2 FN3KRP SMURF1 NIPSNAP3A CCDC69 RNF167 CORO1A C17orf62 PSMB4 KIAA0101 PNMAL1 NAGS LY9 ZNF341 OR2A14 OR1E1 QPCTL RBM6 MIR1179 LOC729082 GLTSCR2 USPL1 GATAD2B WDR82 HIST1H1E CRIP3 FAM103A1 MYT1 OR10G2 HLA-DQB2 NLRC5 C11orf20 DPM3 ZNF468 ARHGDIA RPL23 S100A4 ARF1 ZDHHC24 KIAA0907 SLC41A3 SCARNA2 PRSS42 KRT79 RCN3 TRIT1 C19orf76 ENKUR FCRL5 RAB11A ABI3 LOC728264 ADM2 CYP4F22 RPRD2 GBAP1 PHF5A RAN DDX23 ZNF573 LOC222699 RCAN3 LOC286016 LOC100216545 UGT1A10 COG3 ZBTB32 TMEM105 CBS PPPDE2 MLL5 ZNF193 GMFG ELAC2 VASP GRK6 OAZ2 GRB2 TCTEX1D2 PLA2G16 VTCN1 PKLR H1FX MORN4 HIGD1A WIPF2 KIAA1191 OGFR C6orf15 EPHA8 DIABLO BAHD1 SNORA10 TMEM215 ATPIF1 KIAA0232 KLF1 HIST1H3G DLK1 PCDHA12 MEPCE GABRR2 FAT2 GMIP TULP1 LETM1 CTU1 TUSC4 PDGFA KRR1 PLA2G4A WFDC8 HMOX2 TRPV3 RRAS CHD8 ZNF217 ARL5C PNLIPRP2 H3F3B VPS29 UBR4 DKKL1 DCAF7 SFRS18 C15orf38 ULK4 SLC22A7 IL32 MAP4K1 WDR6 C6orf52 TARBP1 EXD2 SLC26A4 PNPLA7 SPHKAP RNF34 LILRB1 RABEP1 ZSWIM3 PSMB8 H3F3A MRPS15 NCRNA00162 JMJD5 DFNA5 C22orf28 BUB3 GNRHR2 RRP15 ELF3 DCLRE1B C1QTNF1 RPP21 STRADA RPS11 TMOD3 EHD1 EFHD1 ALDH16A1 STAT3 CDC34 PRPF38A PLOD1 NUBP2 SUSD2 FAM41C PLEKHG4 SH3TC1 CSNK2B BCL7A CSNK2A1P LOC100130093 C13orf27 ZNRD1 PLA2G4E HDAC1 PTPRO SPACA1 ANXA7 LGI4 HMGN4 YIF1A SPTY2D1 FADS6 NFE2L2 C14orf64 ABCE1 DPAGT1 DACT3 MESDC1 BATF2 AGRN STEAP3 SLC5A8 SLC4A10 MYEOV2 GAA ZFYVE26 CD84 DUS2L HIST2H2BE EIF4A1 ANAPC11 STYX C1QTNF5 KCTD19 GNAI2 C16orf81 KIAA1244 C14orf180 ADK KBTBD3 ICMT GPRC5C UBXN6 POLRMT ETV3 CCDC151 C5orf45 ZKSCAN5 C7orf68 TRAPPC4 CALCB SPEM1 SUPT5H ETV5 DAPK3 MIR134 GTPBP1 RPL13AP5 CIDEC MORG1 NDUFA9 PCNXL3 CD55 BTBD17 FGD5 SYF2 PHF15 EEF1G PIM3 SNRPA TJP3 UIMC1 TCHP ABCG4 C3orf25 RSRC2 CDK10 KCNN3 NEK2 RIMS3 OVOL1 JAKMIP1 VPS39 ARID5A MIR522 TMEM79 TRIM37 C15orf23 DNAI2 MTMR4 HIST1H2BG MIR147 LOC150185 CCT6A SCLY SCNM1 BLOC1S3 PLEKHH2 FZR1 TRPV1 CBLL1 RBM4 CREBZF EIF4A2 PYDC1 LOC388428 EFNA4 TOB1 TMEM67 KDM3A PANK2 STT3B GALK1 TOP2A KCNG2 TDRD9 TTC30B KIAA0528 HCN1 C22orf24 FITM2 WFDC10B IGFN1 EIF6 C1QTNF9B DNPEP SH3BP1 ZNF595 LOC100130017 STAMBP GNAI3 ATP5G3 GADD45B AMIGO2 C1QTNF2 CMTM4 OGFOD1 RPL10A AARS MED11 NLRP11 SERTAD3 SNORD32B BTF3 RPS12 KDM2A DR1 C20orf85 RELB SUNC1 ZNF235 BCCIP GCDH HIST1H2AL TBC1D8 C3orf45 RNU12 INTS9 TBCB TSPAN12 PNPLA6 KLF17 ADSL EBI3 BRIP1 ATP1B2 DDIT3 RPAIN LOC100132354 GRM6 ZNF114 AGFG2 AQP6 ZFP91 GPR162 KPTN OSBP HLA-DPB1 VAMP4 SLAMF1 MIR451 GARS LOC646851 XRN1 ANGPTL4 ASAP2 SNX3 FOXN4 TOX2 C7orf10 PIM1 KLK4 BCL2L1 ZFYVE27 MIB2 RAB5B TNN SFRS3 ZNF467 TBC1D22A TCTE3 ADH5 MYCL1 MAPRE2 TMEM88 HYAL3 FLJ39582 NUMBL SLC7A6OS G6PC MGC14436 RFX2 SLMAP SYCE2 NAA16 ESR2 GUCY2D KLK11 ZNF585B PNPLA1 FLJ25758 S100A1 CDC27 SH2D2A PVR ZMYM1 RNF126 TPCN2 TNFRSF1B DAND5 TNPO2 A2M NCRNA00095 FANCI ALPP KCNA10 ATL2 PLIN3 EBF2 NDUFA12 DDX5 E2F2 MYCBPAP C1orf43 ASPSCR1 PLA2G1B RAD52 OTX1 SPATA6 MEGF6 PLA2G15 CGREF1 CRAT PRSS48 TNFSF13 POTEA PRLH CEBPZ EEF1D TEKT5 PGLS ZFYVE21 MAS1L MYH16 COX6A1 TLN2 TMEM82 TRAPPC5 LYRM7 THADA MIR219-2 NAPSA WNT6 NDN DDX27 DNAH2 PLA2G2A SLC45A4 FUBP1 TP53 STAT6 C3orf19 SLC25A24 MLN TIGD2 TMEM185B C1orf85 ADAM18 CASKIN2 ISCU GSDMA LOC100133991 WRAP53 PPY AP1G1 IFRD2 KLK10 DENND1A GGCT SH2D4B U2AF2 MIR142 RPL13 PIK3CB PLEKHG3 ILK NLRP4 RNGTT C12orf53 SFTPB ADAM19 ZNF252 C10orf41 THBS2 TM9SF4 GRAMD4 TRPC2 NPSR1 HIST1H4F C8orf46 LAMA3 LOC100189589 MIP CLDN18 LCK C16orf45 FLJ43663 CALM3 ARHGEF19 C17orf95 MIR665 PRKCB TNFAIP2 ARHGEF16 UBE2H USF1 NTNG1 CLIP2 CHCHD1 UBE2L3 SKIV2L WRN C7orf52 CTDSPL MIR192 LRRCC1 FAM174B LY6K MDGA2 U2AF1L4 VPRBP USP37 CACNA2D4 WDR36 ATP5SL INS-IGF2 TACC2 SCGB1D4 PRODH MPV17L2 TMEM69 C6orf136 KCNV2 NFASC PDE11A IER3 C14orf43 MRPS14 CYP2E1 CCNB2 MDH1B OR1B1 BSN WDR91 SLC25A18 HAS3 IVNS1ABP OLIG1 B9D2 EIF4E TMEM161A SLC25A17 TENC1 KIAA1683 MRFAP1 SEC16A LOC90246 TNFAIP8 GPR45 MPP3 PRIM1 MST1P9 SRRT PCDHB13 MIR1908 TMEM106A TNFSF14 GOLPH3L KCNIP1 ZNF540 LHX3 ARHGEF2 RNF111 PANK4 LRFN3 FAM69B LMF2 RAB3A RPL23A FASLG NRTN CD8B WDFY2 NBEAL2 IFI27 SIGLEC8 STAM HFM1 C4orf22 EPHB2 BICD2 LOC728743 SLA2 FBF1 SPINLW1 HCG26 NPAS4 SCGB3A2 ITGB1 LOC153328 ABHD3 MOBKL2A C19orf6 RNPEPL1 PCSK1 CDC25B TOP2B PSMB10 TMBIM1 CAB39 FAM100B ATP6V1B2 CACNB1 MATN4 RAB40C GOLGB1 TIMELESS MYO15A HLA-E PIK3AP1 CPNE5 RANGRF TMEM111 LOC100188947 PPHLN1 KLF11 ATP8B3 ZEB1 NCAPH2 CLIC4 SIM2 HACE1 PCDH24 KIF26A AES TCF3 C1QB CDK5 GNB1L PTPN12 KREMEN2 MRGPRG NGB C13orf36 ZBTB46 TAPBPL ALPK3 SDCCAG3 FAM188B KCNMB3 OR7E37P ZNF766 TFCP2 ITPK1 MYH6 TMEM49 CCDC57 STMN3 CSMD2 NDFIP1 EIF3E CCNG2 EXOC7 C1GALT1 ST6GALNAC1 SNAP23 GDF9 SLITRK2 GTF2B ANTXR1 MMP3 EGLN2 KIF21B BAI1 DCP2 RMRP ZNF689 COL27A1 ZNF121 RP1L1 DHRS7C AHSP FNBP1 MCAM IL2RA ORAI3 SDK2 ADRBK1 RPA1 BAP1 GPR3 PAIP2 HOXB8 RPL22 PADI4 SNORD116-4 SLC30A3 IL4I1 AP3D1 LOXHD1 CDC6 GGT6 KIAA1279 CCDC42B SNX19 PSMD13 ZNF134 PRKAG1 RASD1 LOC100302652 NAP1L5 CLCNKA MIR423 SOCS3 MRGPRF JMJD7 TLCD1 WDR17 ELMOD3 TMEM143 USP7 OR6N1 VPS33A HR PRM2 LRTOMT ADCK4 DDX6 TAF5 SLC25A46 TLE4 DDX60 ARPC1B KY TMX2 CHEK2 FAM83H LRRC4B RLTPR DEDD2 RIOK3 LSS ARFGAP1 UBASH3A C22orf32 PKP3 EPS8L3 RNF19B ZNF284 GOLGA7B LOC641746 CALML3 ZNF143 PLXNC1 C22orf41 SH3GL2 FBXO28 PKMYT1 FAM161A C9orf142 CD5 PEG10 IFT172 LMAN1L RPL28 OBFC1 ITGA1 CYTH3 MUC21 GPRIN1 CCDC154 PTGES3 TESC CLPB ALG10B CAMTA2 CCDC15 PRIC285 C20orf166 IL16 GGT7 RAB17 C1orf31 TREML3 FAM162A TRIM56 LOC100128071 EPX CSF3R OSCP1 SNHG4 CLN3 HIST1H2BD SLC25A35 VIL1 SCN10A CHMP6 TTLL1 ZNF202 ITGA7 FKBP11 SPAG4 APLP1 FANCA PLIN4 PREPL DNAJC27 GPR37L1 ASPDH MYO1G MOGAT2 TRIM41 OR5AU1 CEACAM6 CYMP HELQ SLC1A4 SLA BACE2 PRKAR2B DSTYK STAT1 NOP14 GNA13 ABLIM3 PDE5A FAM127C MUSK UNC5C RTP1 RHOT2 RPS4X MRPL20 BSDC1 ZNF347 ESD KLHL20 CYP1A1 FAM133A UTP15 NDUFA2 MAP3K7IP1 ATP4B MASTL TMEM44 WFIKKN2 ACPP HTR6 CD28 MIR662 PSORS1C3 FKBP1B AOAH B3GNT4 HSPA1A LCE1C MEGF11 WNT1 PTPRCAP SFRS7 C11orf51 CBX1 CYC1 IP6K2 C1orf170 ATF4 CDKN1B C8orf79 GPAA1 AOC3 GATAD2A TTLL12 LY6D NTN1 CITED2 TAF6 PCDH1 PARP10 KIAA0427 TP53AIP1 PKD2L1 EI24 ENPP4 LOC100128788 THEG LRRC8E TSGA14 C19orf40 CD8A NR2C2 IRF2BP2 SLC39A13 SKIL RSPH1 GRP PGLYRP3 FLII RPSA HIST1H3D ANKRD34A ERLIN1 KIAA1826 MLST8 ABCF1 SFMBT2 MGAT4C KLK15 CELSR3 MYNN SLC45A3 NMNAT2 QRICH1 APOC4 NKPD1 GPR37 BCAM TMEM104 STAT2 SH3PXD2B ATXN1 ATP6V1F IL2RG CTR9 FAM189A1 INPP5K GAS5 PTPRS TEX2 TIRAP NDST1 DNMT3L RAB11FIP5 SGPP2 CRELD2 VAMP2 HSD17B3 CXXC1 IGFL4 SNX5 MOBKL2C CDON FAM132A ATP1A2 NARG2 CPXM2 CD63 HSP90AB1 C9orf71 HCFC2 COBRA1 PREB KLHL30 TSPYL6 DCAF16 CHD4 C3orf72 CYP2W1 KIF1B DUSP2 SLC43A2 LIF ZNF577 SEMA7A CAMK1D RPS5 METTL8 BCAR3 ZC3HAV1 ABCB8 PEX13 RPS26 MIR770 CDC42EP3 DENND2C MIR200B CKS1B LIN52 SNRNP27 AP2A1 HLA-C RNF175 SFRS15 HOXD1 CREB3L2 THTPA HBXIP ARID3C NDOR1 C1QA SH2D5 FAM20A SNORA26 MGC16703 FUT5 CCDC134 FAM5B LILRB4 MIDN TSEN54 TBC1D23 C10orf140 HCK TCF7L1 MMP17 APOE SLC17A4 CORO6 HIVEP2 PPIL4 OCLN APH1A C1orf158 C21orf70 NPS TXNRD1 UNK COQ2 ZNF461 VAT1L MDC1 ZNF841 EFNB3 THRAP3 LRCH4 KRT83 PTCHD2 MFSD4 DPF2 CCM2 CDADC1 ACBD5 TFR2 PDE1B ZNF266 CORO7 ZNF441 DENND3 DUSP4 NFYA MYLK2 C5orf35 FAM177B YPEL1 WNT3 IGLL3 C17orf79 C15orf27 HSD17B7 PTPRK TTC3 C6orf201 GRIK4 EVPLL ANPEP NAV3 C22orf39 LSM2 PDCL2 GRTP1 TOMM6 NUPL2 C22orf42 CX3CL1 MYB OR2C1 MUS81 ACCN2 C6orf27 CKAP2L CNTFR ZBED4 C1orf103 GLTSCR1 FLJ40434 PPT1 GABARAP ATP13A2 NECAP2 LRRN2 SLFN11 GIPC3 DISC1 CAPN14 TMEM184A SKP1 PTCRA INTS3 CORO1C SDHC ZNF234 RNMTL1 ALDH1L1 SUV420H2 IKBKE PVRL2 DCBLD2 FFAR2 WBP11 ZNF583 CLDN19 CXCR5 CNKSR3 MAP3K14 PRG2 TCF15 C14orf21 ZFP36L1 TFAP4 LMO1 BTBD19 AGXT ALDH4A1 SPAG1 MC1R CD37 TRIM38 ZP3 ELOVL5 KLC3 SEMA4G SAP30BP AGPAT3 TTLL10 HRAS TSPO PLS1 PBX1 TRAK2 TLR9 S1PR1 ORMDL3 RIMS2 ZNF628 HNRNPL C1orf187 GAL3ST1 PTP4A3 CAP1 STARD3NL C20orf118 WDR51A OR2H1 C3orf31 H19 MIR874 CCR9 SFRS14 C6orf226 RBM5 TMEM187 TRPV5 MIR603 NPTX1 CHRNA3 OR7G2 SMAP2 EFNA1 KCNH7 FAM55D SLC22A23 MAPRE3 IP6K3 CMC1 SLC30A2 PTBP1 IVD CASC1 RHBDL1 HEPACAM GSDMC SNORD87 ARID4A INTS7 SRI PIAS3 HDHD2 FBLN7 FAM135B LPCAT4 C11orf83 C14orf79 C16orf63 ELTD1 GJB3 MFNG CCNJL GNB2L1 ZNF480 MCART3P SVOPL PTPN3 SLFNL1 DECR2 ACOX3 SNTA1 RPS29 WFDC6 CNGA1 FRMD8 NPR1 TSC2 CAPS ODF3 SLC23A1 SFRS2 ABT1 TMEM126B RABGAP1L MBL2 CAPN12 REEP1 ZBTB8B HLA-L SLC39A7 SULT2B1 PNLIPRP1 LOC285456 LIME1 TARS2 PAPD5 FAM179A ZDHHC12 ITGA11 DLEU1 PCDHB8 ZNF562 SF3B5 SMG7 MANEAL E4F1 PYGM SPRR1A C17orf89 TEAD4 LRRC23 PFAS NUDT15 TAGAP CDR2L ASB2 PHF14 PRPF38B OR52N2 OR4F15 TAS1R2 WDR75 TAF10 CD1D FLJ37543 MRPL24 LOC100128675 PYY FURIN SPIB WSCD1 TMEM181 MRPS33 PCBP1 NEURL4 RRM1 ARAP1 SERPINB5 ACAD9 IQCA1 SNRPB PPP1R11 MYL5 HIST1H2BN IMPA2 GPR119 PRR12 RECQL4 ID3 SRL GOLGA7 FARP2 C10orf84 SHMT2 TBL1XR1 ST6GAL1 DMRTC2 DSN1 MICAL2 ELFN2 SBK1 SOCS1 REEP6 ASH2L C7orf34 RNF168 ACADS TBCCD1 DUSP7 SOX12 SUCLA2 OR1F2P EID3 USE1 ADCK5 KIF9 GALM C19orf69 GLTPD1 C1orf35 RNF212 RFX5 HIST1H2BE LGSN SEMG1 VIM PIK3R3 PLAGL1 GABRD TDG CHRNA1 HTR4 ZNF541 RHOV CRYBB3 CENPO SPARC SCNN1B C17orf42 GFRA3 TACR3 ZNF677 SELK GPR116 MFSD1 CCNF WFDC1 MYL6B CYP7B1 PRF1 SLC5A6 PLCD3 CHAD LAMC3 DPP10 ZNF823 B3GAT3 DLGAP4 C19orf48 NIPA2 PODNL1 KRT78 PRDM15 SLC39A8 TNFRSF11A TPM4 LYPD3 DNAJC4 C11orf48 SFRS2IP RAP1GDS1 TREML1 SNX21 UHRF1 C11orf1 NAPRT1 FAM76A PRELID1 DNM1 PUF60 MRPL35 WIZ PPP3CC AP3M1 USP5 GSTTP2 EIF4B PIGO USP35 CACNA1B RHBDD2 AHCTF1 GART ALK SNORD2 PCDHGB1 CRYAA TBXAS1 CSNK1A1P ABCC1 CWC22 LECT1 MOSPD3 FSTL3 NUDT8 KCNJ14 YIPF3 TROAP CCDC47 CEACAM16 TCP1 CFB CCL28 NLRP14 COX15 HSPA5 ARHGDIG MIR1286 ADAM5P C16orf92 ZRANB2 IL22RA1 PPARGC1B WNK2 MAMDC4 CYTH4 ZNF653 CAPN1 MIR1229 CHORDC1 PNP SMARCA5 CACNG5 HIST1H2AG ZNF512 LOC256880 C13orf33 MIR33B EML5 LOC653653 RNF166 PPFIA1 GSTT2B SEMA3B RTDR1 AFG3L1 INSC GPR126 NEURL1B MAPKAPK3 LYNX1 EXOC4 MYH7 PHRF1 ZNF226 GPBAR1 GABRA1 RASSF2 PRKCI MAP3K11 SLC2A9 PTP4A2 BRMS1 PRDM6 LOC285780 EML3 SPRED3 TCAM1 MKL2 GDPD5 HHIPL2 ZC3H7A CLCN3 FLJ40852 MIR641 H3F3C NCCRP1 SF1 FHIT ABCC12 ITPR3 LAMA5 SON PIKFYVE NHEG1 RDH8 ZNF3 C9orf3 OR1M1 SCML4 LOC389458 ARMC5 ETV2 CIB4 NRXN3 HSPBAP1 LPHN3 TMEM50B MYOM3 SUFU C12orf65 DUOX1 SFRS16 C9orf75 UBXN11 ASPA C6orf222 ADORA2A C1orf133 RNASE13 DNMT3B PCDHB12 CAPRIN1 IER3IP1 ALKBH4 KDM5A TRIM69 SLC13A4 C10orf129 BDP1 HES5 COX5B BRSK2 CD3D PRRG2 FAM72A C8orf76 COMTD1 CCDC163P DBNL RAB35 SYT16 OR2H2 RAP1GAP LRRC4C IL2 CAMK2A SIT1 TREH MYL7 PANX2 SULF1 LY6G5B MPL PIAS2 ABCC8 CHST8 DEAF1 SNAP47 TSSC4 C1orf63 PLEKHG5 T-SP1 ZNF345 PPP2R1A EBF4 NAGPA C1orf128 DEXI PRPF3 PRRT4 PLA2G2E LMLN AEBP1 SNORD12B CDK16 DDX39 MTP18 C20orf195 COL17A1 FAM171A2 FIGNL1 SNX29 GPC2 ACRBP LOC146336 ATP5H NME7 GAP43 ZNF678 PSCA C9orf119 RBM34 LTBP3 OR14J1 ATL3 C14orf138 KIAA0415 RMND5B AK2 CCDC132 PITPNC1 SESN2 FBXL16 VIPR1 ACSL5 LAT LOC154449 OR13J1 CD300LB TLR6 MOBKL3 FGF6 MPDU1 SP3 ASCC1 LOC389333 FAM98A ALDOA TRA2A RBL1 LRRC14B SRPK1 MICALCL HTR2A TGM3 COL13A1 SLC27A5 ISM2 NFE2L3 SCAMP3 PPOX FYCO1 DEFB128 LRRC26 TNFRSF25 C7orf65 LOC100271715 OTX2 TMEM8A NUMB PBXIP1 CASKIN1 CIC ILF3 RGS14 FOLR1 KIAA1671 VARS OR56B4 NPLOC4 ZIK1 GPR152 HSPB1 SLC6A20 ISG20 TMEM213 BMP5 VMO1 NHLH1 ZNF282 ATP6V0A2 MFAP5 UQCRH RDH13 C1orf216 LRP2BP PDZRN3 GRID2IP DGKQ THG1L DNAH5 PRR23B MIR655 TARDBP DPYSL5 OPA3 ZBTB11 HNRNPA1 TCTA C2orf18 FAM50B MRVI1 ZBTB5 C4orf44 NEO1 ADAM15 FAM9A CAMKV ZBTB47 RGS5 PLA2G4D CA7 RPS14 C11orf60 HEXIM1 ZNF580 TMEM53 MXD1 TTC31 SPIRE2 C13orf1 SYT3 KIRREL CDKL2 GRINA C10orf93 RUNDC2A DNAJC14 NCAN GANC LOC650226 TMOD1 EPHA6 PLSCR4 FABP1 MAP3K10 CDX1 AZIN1 BLK HMGB2 PPM1K KLHL9 SARDH NPC1L1 ZNF215 FAM192A RAB4A FGF22 ABCC3 SEC24C ZSWIM7 NEU4 RTBDN GLI1 KIAA0652 FGD2 PHF19 HRH2 FDXR MAPK11 LOC84856 CHCHD5 FAM53A CNTD2 PFN2 C14orf177 HNRNPR PSMC3 TMEM65 ECE2 CELSR2 PSMB6 PNMA1 AIM1 NCAPH ZNF767 OBFC2B GAS2 RIC8A LAMP3 UNC84B PRDX1 C11orf94 ADPGK DAB1 PDE1A TUBD1 NIT2 HPCAL1 C15orf58 WDR5 GPATCH2 LOC255025 RAB2B CLEC4D NKAIN3 SNORA64 THSD4 SLC38A4 SIGIRR TADA2B NPHP1 ADCYAP1R1 CCDC146 TUBB2A SERP2 HAPLN3 MIR216A ZNF438 KCNC2 CCT6B TNFSF10 PTGDS IL10RA SUGT1P1 OR2V2 ADAD2 DSPP STX2 CD82 SRMS BSG NRCAM ZSWIM4 AARSD1 SPATC1 FEV SNORD27 MRPL48 HNRNPAB ARHGAP17 GMPR2 C3orf38 SLC25A39 PRMT2 C10orf11 UBL7 PIGH KRT28 MPDZ CRAMP1L REXO1 ZNF317 RBM14 HIST1H4D GNG8 SYT8 CNN1 OLFM2 C11orf58 PP14571 PPM1G PPP2R2C TMF1 MAP2K5 LIMS1 GIPC1 MRAP2 CCKBR C3 LOC96610 SDCBP2 HES7 ENDOG SMOC1 KATNB1 FLOT2 C6orf211 FAM173A TRIM62 TNNT2 RAPGEF5 C1orf111 GSK3B PIK3R6 SFPQ RPL24 TMEM41B C17orf93 C20orf111 MIR29B1 TM2D2 SIRT3 CBX6 DENND4B GUCA2B ZNF542 ROPN1 UNC84A GPR179 DAPK2 SIRT2 OR12D3 YDJC EFS PCDHB19P SART1 RAD51AP1 ZNF498 ZCCHC4 DGCR10 CACNG4 ZFP36 C2orf71 SMARCD2 COX8A SSPO LOC100169752 TMEM63C RXRB DDX18 FBXL14 PTPN6 ALS2CL CREB3L3 ANKRD24 MGC15885 FBXL15 MALAT1 CHST15 ZSCAN22 ZNF683 ENHO C1orf88 BBC3 TRIM58 SOCS7 ZNF133 RUNX1 FAM96A SNORA63 CBR1 RYR2 GUCA2A EDC3 SNORD105 CDH23 PCDHB17 RNF139 HCST PRMT5 SLC4A1AP PTH1R SNHG3-RCC1 HMG20B NDUFV3 PFKM MED15 CNTN4 SLC39A6 ERN1 CD226 USP29 MIR1289-2 KCNK7 C6orf134 SLC26A9 SLC1A1 FA2H HABP2 IL2RB ZNF805 MATN1 YEATS4 C12orf34 MCM5 TBC1D9 DCAF4L1 ZNF323 STAM2 MRPS21 NAAA SLC25A16 FXC1 RIT1 UTS2R MRPL55 ANKRD13A TRIM45 CENPA HLX TACSTD2 LOC55908 TMED10 BPI ZNF264 NID1 HSPD1 ANKRD13B MTERFD2 SMAD1 DDX42 FGF23 PRKAB2 CD19 ATP13A5 PDGFRB CCDC87 RABGAP1 APLNR LAPTM5 ELFN1 OR10X1 APOA4 SEPN1 RPL5 NBR2 GDF15 EDNRA RPS6KB2 ATG16L1 DPP4 CLK3 ZNF222 PIK3R5 GJB2 LOC202181 CEACAM4 CNTNAP3 XYLT2 SCGB2A1 PFDN6 EEF2 CDKL5 DRG1 POLR1A TLE1 GPR78 C6orf35 RCCD1 SEPT1 SEPT9 SEPT10 FAM163A IGSF21 MAGI2 MTSS1 TRIM26 HOXC4 ODF3L2 TNRC6C DEDD PALM NFIC PTPRG PTPRN2 ODZ2 ST5 KCNQ1OT1 LYST SPOCD1 AFAP1 DLG2 PRKAR1B DOCK9 RUNX3 GRAMD1B SYNGAP1 MUC2 MAP4 KIAA0319L HCCA2 KIAA0513 SAPS2 ACHE COL9A3 IGF1R MACF1 HRH3 CD81 CD3G FOXP1 PIP5K1C HOXA3 APITD1 RAPGEF6 TNXB TIMP2 MAPK15 RERE NFAM1 VPS13D TNRC18 ANO1 RPTOR P2RY2 MYT1L KDM2B NEB BAHCC1 ATXN7L1 ETS1 FCGRT PHF21A ADARB2 CENPP TBC1D16 PRDM16 SLC12A7 TLE2 SND1 AP2A2 GRK5 ZFPM1 MAD1L1 COL5A1 TNIK SLC13A5 BRUNOL5 NRXN1 ARRB1 CCDC55 RNPC3 GALNTL6 PLEC1 BAT2 ZNF238 GRAMD2 ZDHHC14 NLGN1 DHX16 SLC9A8 JOSD1 AGAP1 EXOC2 KCNQ1 CACNA1C ARHGAP10 GNAS ZC3HAV1L TJAP1 ANKRD11 C15orf33 AHDC1 HOXA10 RNF39 FAM131B DPCR1 UST MYST1 RBMS1 NF1 COL22A1 GNA12 MBOAT7 CCDC92 OSBPL5 CADM2 MRPS16 SPATS2L UNC93B1 TTC7B ACOT7 SBNO2 NR3C1 MTUS2 GAL3ST2 TP73 MIR548H4 ARID3A MXI1 ZAP70 CAMTA1 RBM47 ARIH1 MGMT MGC34034 FAM101A LOC404266 KCNMA1 PITX2 C1orf86 ACACA KDM4B PTRH2 B3GNTL1 GLP2R SKI MUC5B BCL3 PCGF3 CENPL SGEF CNTN2 ZNF420 ARHGEF17 C19orf38 SHANK2 NHEDC2 SLC9A3 EVI5L LSM4 NCALD PLEKHN1 ZNF423 KNCN PRR3 CLK4 SMCP HIVEP3 GATA4 TTYH3 CUX1 THOP1 LAMA2 JPH3 CLEC11A DMRTB1 RING1 BTNL2 BAIAP2 LARS2 WDR60 MCF2L2 IREB2 TEX14 RASAL2 DNMT3A MEST TCF7L2 RASA3 NIPSNAP3B TNK1 RBPMS KLHL29 FAM172A DPYD TTC18 CDK6 IFT52 WBSCR16 CBX2 SDC3 EXD3 LAD1 MARS2 NAV1 CBFA2T3 OTOP3 RTN4 NOTCH3 NFIX DYNLT1 MAML3 MTG1 RPL41 CALY HS3ST3B1 SLC12A4 KLC2 NOS3 GNG2 PHC1 LOC283999 DIP2C BCL11B DOCK1 MIIP HEATR2 C7orf50 GBX1 STK39 SGTA KIAA1688 ITGB5 ATPGD1 SLC44A2 FAM102A NUMA1 C1orf51 MAP4K4 PPM1L NBEA RGS12 LOC100130987 PRKCH HOXB7 CCDC108 PRKCZ ERI3 PRPF8 CLIC1 COL4A1 PNPLA2 AGER SLC22A11 SOX2OT RGL2 PELI3 HMGN3 ANXA6 EPHX1 KCNQ2 KIAA1751 ZBTB12 KIAA0182 DTNBP1 AHRR MAEA BZW2 RIMBP2 CLUAP1 PEX14 NADSYN1 TIGD3 SPI1 GALNS GPR4 VGLL4 IQCE CCDC46 TRIO CAMK2B KIAA1026 DOCK2 CCNDBP1 TNNI2 ECHDC3 NOTCH4 PPP2R2B C1orf101 NCRNA00171 HS3ST1 NES CALD1 MYH10 MYOCD C8orf73 SLC22A18 RREB1 ATP11A LRP5 ARMC7 GABBR1 RADIL GRIN2D MDFI ARFIP1 SLC38A1 C6orf106 GALNT9 SFRS8 ZBTB41 UNC5B PITPNM2 IGSF22 DSCAML1 PARD6A FRMD4A CCDC88C TERT FLOT1 DLC1 SETD2 CHD5 RASSF5 SLC16A5 FAM176B TRAF3IP1 KLHL26 NAV2 FXYD1 MAP2K1 ACSL3 PTPN23 RILPL2 ADAMTS2 VSX2 JAK3 MAN1C1 DAXX SEC61A1 RPS6KA2 ZNF10 ORMDL2 LBH AKAP13 PDLIM4 ZNF148 CYP11A1 FBXL7 ACSF3 STK32C HAPLN4 CSMD1 KCNQ4 ANKS1B ATP6V0A1 TMEM89 SSX2IP COMT FAM115A KIF7 EDAR FAM155A KLF3 WDR46 AMPD2 LY6G5C RAB7A LMNA SOX1 GMEB2 ATP9A AHNAK PCDHGA4 STAB1 CACNA1H IFFO1 HCG9 GFAP DDX54 PC SLC16A3 TSPAN14 SDK1 CARD14 PRKAG2 RBMS3 TULP4 HERC1 RAP1GAP2 PLVAP ANGPT1 EMP3 FAM38A SLC44A4 PITRM1 CS RAB1B OCA2 ETV6 CTSZ PARD3B KPNA2 SLC38A10 C6orf89 KCNK3 PHACTR2 LASS4 EIF4G1 FBRSL1 SLC9A1 GRIN1 KIF1C NPR3 SLC5A9 SHC2 CDC42BPB MTF1 TNPO1 EP400 TPPP TNS1 RNH1 GNG13 SLC6A3 INPP5A ANKFY1 MRC2 KCNJ10 TAP2 INO80D C9orf171 FNDC3B C9orf25 SNED1 C1orf159 GRK4 PPARA KIAA1324 KIFC1 SCARA5 KCNA3 MAST1 NBLA00301 DIRAS1 PGCP DOCK4 MEIS2 LRRK1 C1orf175 MAPK10 SLC22A17 PLEKHB1 C19orf35 MKX TRPM5 CHPF2 OSBP2 ANK2 EYA4 LENG8 PEG3 SLIT3 SHANK3 ZMIZ1 C6orf48 MMP15 TRIM2 AMOTL1 COL5A3 CALCOCO2 C9orf173 SEC22A HRNBP3 NECAB1 COL6A2 BANP MEOX1 CGN DGKH IL12RB2 FHL3 PRSS33 KIAA0284 TRIM40 SHANK1 FZD5 ACCN1 AMPH HCRTR1 C8orf33 COMMD2 SYT7 PER1 IGSF9 MYO9B LYSMD2 TGFBR3 SHISA9 COIL TMC8 ZNF644 ATF6B EHMT2 FAM107B UQCR SEMA3A PHYHD1 EVL ZBTB7A RAB8B CAPN5 C5orf4 C21orf29 COL1A1 POFUT2 COL11A2 B3GAT1 DNASE1L2 ZNF783 HOXC9 ARHGEF10 B3GNT3 CAPN9 PPP1R9A RPAP1 COL6A3 BRUNOL4 FAM131A GPR153 SLC12A5 CD44 F10 IL28RA MAP7 SGCE MAP3K8 SOX6 NDUFS2 CREBBP RNF220 SRRM4 ITGB2 ASPG RAMP1 OTX2OS1 PPP1R3E KCNJ6 LRRC32 PELI1 ST3GAL4 SH3RF3 AP2S1 ITPKB TRIM31 TEAD1 PLK5P FOXK1 TCERG1L SEC14L1 KCND3 C21orf33 CACNA1I TNFRSF8 CNGB1 LMF1 NPHP4 HERPUD2 PLIN1 POU6F1 ADAMTS13 TBC1D14 ZC3H12A IKZF5 AHR UGP2 HLA-DMA SLC4A2 TRIM39 TCTE1 C5orf42 NDUFS8 PRLR RRAS2 GNG7 PACRG FANK1 CACNA1A DUSP10 LTB SLC30A7 DOK3 RFESD ZC3H18 MAPK8IP1 PCDHA2 CCDC67 AGAP3 C7orf20 KNDC1 HLA-J SLC22A6 LSP1 C10orf82 CLEC4C AGPAT1 SORCS2 ADCY4 TMEM183A MFSD7 CXXC5 CTBP2 NEUROG1 RASGEF1A GNL1 TRAF6 DYNC1H1 RORA DENND4A FNIP1 P2RX1 CRTAC1 PHLDA2 ZBTB22 FGF19 PLCH2 THAP2 AGPAT2 OTUB1 GRID1 HLA-DRA MDGA1 PLAT CLSTN3 FGD6 GNASAS CACNA2D3 DGKE HIF3A FAM125B HIPK4 SRRM3 GLI3 S100A16 ZBTB2 ITPKA OGDH NRM SPTBN4 PAX8 IQSEC3 CDK14 MEGF8 C20orf117 JMJD6 FBXL18 HLA-F VARS2 NXN EIF4E1B SLC9A3R2 FNDC3A EIF2C2 AATF RYR1 SETD7 CTTN TATDN1 STAU2 RASGRP1 FHDC1 IFT140 CHST11 ASCL2 PCDH21 NTM SEMA4B FAAH TMEM217 SHC1 PTRF PGD ZC3H3 TRPC7 AXIN2 ST7L BAT3 SCOC ANO6 ADAMTS10 STARD3 RPH3AL C10orf71 ADSSL1 NTRK3 KIF13A ADAP1 POLR1C STEAP2 C6orf114 COL7A1 CCBE1 FCGBP VEPH1 ANK1 EGR3 PSORS1C1 METTL9 RFX1 XKR6 GNG4 USP36 NFKB1 SLC1A2 SLC22A3 PDE4A MYBPC2 FTL PSMA1 NRP1 SIDT1 LPCAT1 CARS2 C5orf13 RHOBTB1 C3orf50 COL18A1 WIPF3 ZNF763 SFXN5 BAT1 BTBD12 ELF1 ARRDC2 TIA1 CLSTN1 ZNF556 AGBL3 RINL FAM19A2 SCHIP1 PMEPA1 CCDC85B KIAA1462 AAK1 TRAPPC9 ZNF365 BRE TSPAN9 SEMA6C LLGL2 PPAP2A MCF2L CHL1 CDC14A PRSS22 ULK1 POLG FASTK RPS9 KCNH3 GTDC1 ZNF469 STK24 MYRIP MZF1 SNORD116-17 MSI2 SLC8A3 COL6A6 CDKN1A RFX7 KPNA6 MLPH GAK RICTOR TNNT3 PIGV AHCY VAV2 DBP CHRNB2 NXPH1 MACROD1 TH FAM20C BRF1 TTF2 DOT1L OBSL1 MIS12 ASPRV1 FGF14 LRIG1 NIPAL3 GRIN2B MAPK12 TRIM15 HNRNPM ZNF354A CASP3 SSU72 HIC1 MOV10L1 TNK2 SLC39A4 BRD4 RGL1 SLITRK5 CHID1 ARF4 ALDH3B1 ZNF614 ENPP6 C10orf53 CCDC48 EFCAB6 RAB3C CDYL SUCLG2 NRXN2 SCN4A SCARF1 ACTN3 C9orf68 PTTG1IP CUX2 MEFV LRP1 MYOZ3 KIF3C CD300C OS9 LOC339524 CYFIP1 KCNB2 SHF PLEKHO2 AUTS2 PTPRE SLC2A5 SUCNR1 ATP4A GRM2 PAK6 CDC42BPA BMPR1B PCSK6 CAPZB INPP5D C10orf96 NOL4 P4HA1 SCD PLXNA3 NR5A1 UNKL MSLNL RALGPS1 C10orf90 UNC13A KIAA0556 EPB41 GRIA1 VTI1B NKTR CAMKK1 CRHR1 PLEKHG4B WWC1 C14orf181 PLA2G2F S100A11 ZCCHC14 KSR1 ADAMTS17 LRRC27 C22orf33 CDK18 CDH13 MTHFD1L RASGEF1C ZFC3H1 CACNG8 GRM4 NHSL2 HIPK2 DEGS2 LPGAT1 CMAS SSTR4 CALN1 LEMD1 ZNF565 NHLRC4 CMIP DNAJB6 TSKU KCNS1 PLXNA1 APOC3 NKX6-3 PHLDB1 DDC ODZ4 C6orf108 SSC5D RHBDL3 SULT1A1 FMNL3 C3orf64 SH2D3A AATK SEMA4A XIRP1 PTK6 CAV1 KLHL6 PIGQ C11orf41 BNIP3 MST1R LGR6 MPRIP ISYNA1 HOXC12 EZR WDR27 CLPS ZNF706 RAPSN ASCL4 FLYWCH2 GGPS1 SETBP1 ZNF827 RDH12 INPP5B IL27RA TBX15 MED24 DIP2B CCDC78 IQCG HDLBP CECR6 CFD LGALS3 CASZ1 GPR176 DLX5 IFNGR1 IKZF3 KDM6B PAPLN UAP1L1 SCAND3 DPP6 GDF1 ZNF385A GRIK5 SLC12A8 TRPM4 WDR37 DUSP16 GOLIM4 FILIP1 RUSC1 C12orf72 CAPN2 CTLA4 SAMD4A CBX4 ABCG8 SLC35F1 SEC22B JSRP1 ISLR ANKFN1 SCNN1D IMMT CRADD LDB1 PIP5K1A RPS15AP10 TRRAP ECE1 ARID1A SH3PXD2A DENND2A MARK2 TDH UVRAG GPSM3 KCTD11 P2RX5 MYADML2 PLEKHA6 HMCN1 RHBDF2 SOX13 ST6GAL2 TET3 SLC4A11 VPS53 WHSC1 SLITRK1 TMEM131 ZEB2 GNS GALNTL4 SLC7A14 SPATA20 DNAJC8 ZBED5 NODAL SRRM2 TFAP2D ADORA1 PLXNA4 DOK7 MKS1 STRN MAP1B UBE2W SH3BP4 HSD17B13 VPS13B ERBB2 SRCIN1 EFNA2 ABLIM2 DHRS7B BICD1 VGLL2 VPS45 DRD2 LTB4R SCRT1 MGEA5 CNOT6L PTDSS2 PIGR PHF21B ONECUT3 ENO1 SLC15A1 CHRM2 C6orf176 EFNB2 FAM45B RNF182 LPAR5 ROR1 STK19 CYFIP2 BTBD11 KCNIP2 PCNT CPA5 POLQ LY6G6C AFF3 MRPS18B MED13L LIM2 CDH22 TXLNA ARHGEF10L SETX ABTB2 C21orf57 PNPO SPNS1 TMEM167B SPOCK2 TEAD3 ANGPT4 C15orf52 C6orf10 ADAM32 PLXNB2 S100PBP RBPMS2 CATSPER1 KCNJ9 PPP2R5C C20orf24 TTC26 ZNF331 AKAP8L MIPEP STXBP5 COBLL1 ABHD2 WTIP FXR2 CXCR4 USH1C PLXDC1 LOC100133545 HPS4 ZFYVE28 SDF4 LOC283663 NEU1 PPME1 SCTR PAFAH2 SLC45A1 NRG1 |

**Table S5.** KEGG pathways of the 46 hypo-up genes are involved in

| Number Pathway terms |  |
| --- | --- |
| \| 1 \| hsa01100 Metabolic pathways - Homo sapiens (human) \| \| --- \| --- \| \| 2 \| hsa05165 Human papillomavirus infection - Homo sapiens (human) \| \| 3 \| hsa04151 PI3K-Akt signaling pathway - Homo sapiens (human) \| \| 4 \| hsa04810 Regulation of actin cytoskeleton - Homo sapiens (human) \| \| 5 \| hsa04015 Rap1 signaling pathway - Homo sapiens (human) \| \| 6 \| hsa04510 Focal adhesion - Homo sapiens (human) \| \| 7 \| hsa04512 ECM-receptor interaction - Homo sapiens (human) \| \| 8 \| hsa05323 Rheumatoid arthritis - Homo sapiens (human) \| \| 9 \| hsa04514 Cell adhesion molecules (CAMs) - Homo sapiens (human) \| \| 10 \| hsa04973 Carbohydrate digestion and absorption - Homo sapiens (human) \| \| 11 \| hsa00790 Folate biosynthesis - Homo sapiens (human) \| \| 12 \| hsa04978 Mineral absorption - Homo sapiens (human) \| \| 13 \| hsa05414 Dilated cardiomyopathy (DCM) - Homo sapiens (human) \| \| 14 \| hsa05222 Small cell lung cancer - Homo sapiens (human) \| \| 15 \| hsa00511 Other glycan degradation - Homo sapiens (human) \| \| 16 \| hsa05202 Transcriptional misregulation in cancer - Homo sapiens (human) \| \| 17 \| hsa04672 Intestinal immune network for IgA production - Homo sapiens (human) \| \| 18 \| hsa04742 Taste transduction - Homo sapiens (human) \| \| 19 \| hsa04611 Platelet activation - Homo sapiens (human) \| \| 20 \| hsa00310 Lysine degradation - Homo sapiens (human) \| \| 21 \| hsa04910 Insulin signaling pathway - Homo sapiens (human) \| \| 22 \| hsa04145 Phagosome - Homo sapiens (human) \| \| 23 \| hsa04360 Axon guidance - Homo sapiens (human) \| \| 24 \| hsa05144 Malaria - Homo sapiens (human) \| \| 25 \| hsa04740 Olfactory transduction - Homo sapiens (human) \| \| 26 \| hsa04744 Phototransduction - Homo sapiens (human) \| \| 27 \| hsa04142 Lysosome - Homo sapiens (human) \| \| 28 \| hsa04918 Thyroid hormone synthesis - Homo sapiens (human) \| \| 29 \| hsa04668 TNF signaling pathway - Homo sapiens (human) \| \| 30 \| hsa05418 Fluid shear stress and atherosclerosis - Homo sapiens (human) \| \| 31 \| hsa05215 Prostate cancer - Homo sapiens (human) \| \| 32 \| hsa04657 IL-17 signaling pathway - Homo sapiens (human) \| \| 33 \| hsa00730 Thiamine metabolism - Homo sapiens (human) \| \| 34 \| hsa00531 Glycosaminoglycan degradation - Homo sapiens (human) \| \| 35 \| hsa04080 Neuroactive ligand-receptor interaction - Homo sapiens (human) \| \| 36 \| hsa00600 Sphingolipid metabolism - Homo sapiens (human) \| \| 37 \| hsa04725 Cholinergic synapse - Homo sapiens (human) \| \| 38 \| hsa05410 Hypertrophic cardiomyopathy (HCM) - Homo sapiens (human) \| \| 39 \| hsa05200 Pathways in cancer - Homo sapiens (human) \| \| 40 \| hsa04060 Cytokine-cytokine receptor interaction - Homo sapiens (human) \| \| 41 \| hsa00230 Purine metabolism - Homo sapiens (human) \| \| 42 \| hsa04640 Hematopoietic cell lineage - Homo sapiens (human) \| \| 43 \| hsa05412 Arrhythmogenic right ventricular cardiomyopathy (ARVC) - Homo sapiens (human) \| |  |

**Table S6.** KEGG pathways of the 71 hyper-down genes are involved in

| Number Pathway terms |  |
| --- | --- |
| \| 1 \| hsa01100 Metabolic pathways - Homo sapiens (human) \| \| --- \| --- \| \| 2 \| hsa05168 Herpes simplex virus 1 infection - Homo sapiens (human) \| \| 3 \| hsa04080 Neuroactive ligand-receptor interaction - Homo sapiens (human) \| \| 4 \| hsa04061 Viral protein interaction with cytokine and cytokine receptor - Homo sapiens (human) \| \| 5 \| hsa05320 Autoimmune thyroid disease - Homo sapiens (human) \| \| 6 \| hsa05321 Inflammatory bowel disease (IBD) - Homo sapiens (human) \| \| 7 \| hsa04060 Cytokine-cytokine receptor interaction - Homo sapiens (human) \| \| 8 \| hsa05418 Fluid shear stress and atherosclerosis - Homo sapiens (human) \| \| 9 \| hsa04020 Calcium signaling pathway - Homo sapiens (human) \| \| 10 \| hsa00260 Glycine, serine and threonine metabolism - Homo sapiens (human) \| \| 11 \| hsa05200 Pathways in cancer - Homo sapiens (human) \| \| 12 \| hsa04658 Th1 and Th2 cell differentiation - Homo sapiens (human) \| \| 13 \| hsa00760 Nicotinate and nicotinamide metabolism - Homo sapiens (human) \| \| 14 \| hsa04659 Th17 cell differentiation - Homo sapiens (human) \| \| 15 \| hsa04310 Wnt signaling pathway - Homo sapiens (human) \| \| 16 \| hsa04062 Chemokine signaling pathway - Homo sapiens (human) \| \| 17 \| hsa03010 Ribosome - Homo sapiens (human) \| \| 18 \| hsa05416 Viral myocarditis - Homo sapiens (human) \| \| 19 \| hsa04810 Regulation of actin cytoskeleton - Homo sapiens (human) \| \| 20 \| hsa04728 Dopaminergic synapse - Homo sapiens (human) \| \| 21 \| hsa04612 Antigen processing and presentation - Homo sapiens (human) \| \| 22 \| hsa04114 Oocyte meiosis - Homo sapiens (human) \| \| 23 \| hsa00534 Glycosaminoglycan biosynthesis - heparan sulfate / heparin - Homo sapiens (human) \| \| 24 \| hsa05330 Allograft rejection - Homo sapiens (human) \| \| 25 \| hsa00982 Drug metabolism - cytochrome P450 - Homo sapiens (human) \| \| 26 \| hsa05166 Human T-cell leukemia virus 1 infection - Homo sapiens (human) \| \| 27 \| hsa04721 Synaptic vesicle cycle - Homo sapiens (human) \| \| 28 \| hsa05152 Tuberculosis - Homo sapiens (human) \| \| 29 \| hsa05323 Rheumatoid arthritis - Homo sapiens (human) \| \| 30 \| hsa01200 Carbon metabolism - Homo sapiens (human) \| \| 31 \| hsa04919 Thyroid hormone signaling pathway - Homo sapiens (human) \| \| 32 \| hsa00510 N-Glycan biosynthesis - Homo sapiens (human) \| \| 33 \| hsa03420 Nucleotide excision repair - Homo sapiens (human) \| \| 34 \| hsa04520 Adherens junction - Homo sapiens (human) \| \| 35 \| hsa05235 PD-L1 expression and PD-1 checkpoint pathway in cancer - Homo sapiens (human) \| \| 36 \| hsa00630 Glyoxylate and dicarboxylate metabolism - Homo sapiens (human) \| \| 37 \| hsa05162 Measles - Homo sapiens (human) \| \| 38 \| hsa05169 Epstein-Barr virus infection - Homo sapiens (human) \| \| 39 \| hsa04514 Cell adhesion molecules (CAMs) - Homo sapiens (human) \| \| 40 \| hsa00513 Various types of N-glycan biosynthesis - Homo sapiens (human) \| \| 41 \| hsa00533 Glycosaminoglycan biosynthesis - keratan sulfate - Homo sapiens (human) \| \| 42 \| hsa03430 Mismatch repair - Homo sapiens (human) \| \| 43 \| hsa05031 Amphetamine addiction - Homo sapiens (human) \| \| 44 \| hsa05218 Melanoma - Homo sapiens (human) \| \| 45 \| hsa04137 Mitophagy - animal - Homo sapiens (human) \| \| 46 \| hsa03008 Ribosome biogenesis in eukaryotes - Homo sapiens (human) \| \| 47 \| hsa04070 Phosphatidylinositol signaling system - Homo sapiens (human) \| \| 48 \| hsa04064 NF-kappa B signaling pathway - Homo sapiens (human) \| \| 49 \| hsa00790 Folate biosynthesis - Homo sapiens (human) \| \| 50 \| hsa05140 Leishmaniasis - Homo sapiens (human) \| \| 51 \| hsa00590 Arachidonic acid metabolism - Homo sapiens (human) \| \| 52 \| hsa05322 Systemic lupus erythematosus - Homo sapiens (human) \| \| 53 \| hsa04940 Type I diabetes mellitus - Homo sapiens (human) \| \| 54 \| hsa03030 DNA replication - Homo sapiens (human) \| \| 55 \| hsa00740 Riboflavin metabolism - Homo sapiens (human) \| \| 56 \| hsa04918 Thyroid hormone synthesis - Homo sapiens (human) \| \| 57 \| hsa00983 Drug metabolism - other enzymes - Homo sapiens (human) \| \| 58 \| hsa00770 Pantothenate and CoA biosynthesis - Homo sapiens (human) \| \| 59 \| hsa05150 Staphylococcus aureus infection - Homo sapiens (human) \| \| 60 \| hsa00980 Metabolism of xenobiotics by cytochrome P450 - Homo sapiens (human) \| \| 61 \| hsa05332 Graft-versus-host disease - Homo sapiens (human) \| \| 62 \| hsa00230 Purine metabolism - Homo sapiens (human) \| \| 63 \| hsa00480 Glutathione metabolism - Homo sapiens (human) \| \| 64 \| hsa05145 Toxoplasmosis - Homo sapiens (human) \| \| 65 \| hsa00240 Pyrimidine metabolism - Homo sapiens (human) \| \| 66 \| hsa04010 MAPK signaling pathway - Homo sapiens (human) \| \| 67 \| hsa04014 Ras signaling pathway - Homo sapiens (human) \| \| 68 \| hsa05226 Gastric cancer - Homo sapiens (human) \| \| 69 \| hsa05010 Alzheimer disease - Homo sapiens (human) \| \| 70 \| hsa05204 Chemical carcinogenesis - Homo sapiens (human) \| \| 71 \| hsa03410 Base excision repair - Homo sapiens (human) \| \| 72 \| hsa04726 Serotonergic synapse - Homo sapiens (human) \| \| 73 \| hsa05224 Breast cancer - Homo sapiens (human) \| \| 74 \| hsa04650 Natural killer cell mediated cytotoxicity - Homo sapiens (human) \| \| 75 \| hsa04640 Hematopoietic cell lineage - Homo sapiens (human) \| \| 76 \| hsa00970 Aminoacyl-tRNA biosynthesis - Homo sapiens (human) \| \| 77 \| hsa00500 Starch and sucrose metabolism - Homo sapiens (human) \| \| 78 \| hsa04068 FoxO signaling pathway - Homo sapiens (human) \| \| 79 \| hsa04015 Rap1 signaling pathway - Homo sapiens (human) \| \| 80 \| hsa05012 Parkinson disease - Homo sapiens (human) \| \| 81 \| hsa04151 PI3K-Akt signaling pathway - Homo sapiens (human) \| \| 82 \| hsa04672 Intestinal immune network for IgA production - Homo sapiens (human) \| \| 83 \| hsa05225 Hepatocellular carcinoma - Homo sapiens (human) \| \| 84 \| hsa00140 Steroid hormone biosynthesis - Homo sapiens (human) \| \| 85 \| hsa00350 Tyrosine metabolism - Homo sapiens (human) \| \| 86 \| hsa05014 Amyotrophic lateral sclerosis (ALS) - Homo sapiens (human) \| \| 87 \| hsa05131 Shigellosis - Homo sapiens (human) \| \| 88 \| hsa05310 Asthma - Homo sapiens (human) \| \| 89 \| hsa00562 Inositol phosphate metabolism - Homo sapiens (human) \| \| 90 \| hsa04371 Apelin signaling pathway - Homo sapiens (human) \| \| 91 \| hsa05164 Influenza A - Homo sapiens (human) \| \| 92 \| hsa05034 Alcoholism - Homo sapiens (human) \| \| 93 \| hsa01524 Platinum drug resistance - Homo sapiens (human) \| \| 94 \| hsa04145 Phagosome - Homo sapiens (human) \| \| 95 \| hsa05202 Transcriptional misregulation in cancer - Homo sapiens (human) \| \| 96 \| hsa05030 Cocaine addiction - Homo sapiens (human) \| \| 97 \| hsa04913 Ovarian steroidogenesis - Homo sapiens (human) \| |  |
